# Cartilage Oligomeric Matrix Protein promotes epithelial-mesenchymal transition by interacting with Transgelin in Colorectal Cancer

**DOI:** 10.7150/thno.44456

**Published:** 2020-07-09

**Authors:** Weilong Zhong, Huiqin Hou, Tianyu Liu, Shuai Su, Xiaonan Xi, Yusheng Liao, Runxiang Xie, Ge Jin, Xiang Liu, Lanping Zhu, Hongxia Zhang, Xueli Song, Cheng Yang, Tao Sun, Hailong Cao, Bangmao Wang

**Affiliations:** 1 Department of Gastroenterology and Hepatology, General Hospital, Tianjin Medical University, Tianjin Institute of Digestive Disease, Tianjin 300052, China.; 2 State Key Laboratory of Medicinal Chemical Biology and College of Pharmacy, Nankai University, Tianjin 300350, China.; 3 Basic medical research center, Tianjin Medical University, Tianjin 300052, China.; 4 Department of Gastroenterology, The Central Hospital of Wuhan, Tongji Medical College, Huazhong University of Science and Technology, Wuhan 430014, China.; 5 Tianjin Key Laboratory of Early Druggability Evaluation of Innovative Drugs and Tianjin Key Laboratory of Molecular Drug Research, Tianjin International Joint Academy of Biomedicine, Tianjin 300450, China.

**Keywords:** Colorectal cancer, EMT, COMP, TAGLN, Chrysin

## Abstract

**Background and Purpose:** The role of the cartilage oligomeric matrix protein (COMP) in epithelial-mesenchymal transition (EMT) in tumor progression has been studied, but its exact regulatory mechanism remains unknown.

**Methods:** The interaction between COMP and the actin-binding protein transgelin (TAGLN) was identified by interaction protein prediction and co-immunoprecipitation and verified through the stochastic optical reconstruction microscopy (STORM) and duolink experiments. Western blot and immunofluorescence analyses were conducted to detect the changes in EMT-related markers after COMP overexpression and knockdown. Molecular docking and Biacore of the interaction interface of COMP/TAGLN revealed that Chrysin directly targeted COMP. The promotion of COMP and the Chrysin inhibition of EMT were detected through the cell migration, invasion, apoptosis, and xenotransplantation of nude mice.

**Results:** COMP interacts with TAGLN in EMT in colorectal cancer to regulate cytoskeletal remodeling and promote malignant progression. COMP is highly expressed in highly malignant colorectal cancer and positively correlated with TAGLN expression. COMP knockdown can inhibit colorectal cancer metastasis and invasion, whereas COMP overexpression promotes EMT in colorectal cancer. Through virtual screening of the protein interaction interface, Chrysin, a flavonoid compound extracted from *Oroxylum indicum*, was found to have the highest docking score to the COMP/TAGLN complex. Chrysin inhibited COMP, thereby preventing EMT and the malignant progression of colorectal cancer.

**Conclusions:** This study illustrated the role of COMP in EMT and suggested that COMP/TAGLN may be a potential tumor therapeutic target. Chrysin exhibits obvious antitumor effects. This work provides a preliminary antitumor therapy to target COMP or its interaction protein to inhibit EMT.

## Introduction

Colorectal cancer (CRC), which has the highest incidence in the age group of 40-50 years, is one of the most common malignant tumors worldwide. Approximately 8 million new cases occur globally every year, accounting for 10%-15% of all malignant tumors 1. Colorectal cancer has a high mortality rate, and its incidence is increasing yearly 2. Therefore, the extensive attention of the medical community is necessary.

The cartilage oligomeric matrix protein (COMP) contains five subunits of homologous polymeric glycoprotein, each of which have a molecular weight of 87 kD 3, 4. COMP, a platelet response protein (thrombospondin, TSP), is the fifth member of the family (also called TSP5) and has a variety of biological functions. COMP can reportedly prevent apoptosis by inducing the expression of apoptosis inhibitors 5-8. COMP is also a prognostic factor and biomarker of colon cancer and promotes cell proliferation by activating the Akt pathway 9, 10. In addition, COMP initiates cancer stem cells by activating Jagged1-Notch3 signaling 11. As a secreted protein, COMP can promote tumor EMT, but its mechanism is unclear 12, 13. In-depth disclosure of the molecular mechanism that affects the expression of COMP protein in colorectal cancer tissues can provide a theoretical basis for understanding the occurrence and development of colorectal cancer.

Transgelin (TAGLN) is an actin-binding protein that regulates the dynamics of the actin cytoskeleton and promotes the aggregation of G-actin to F-actin 14. Our previous studies have shown that excited TAGLN-actin regulates the cytoskeleton and promotes cell contraction 15. TAGLN is recognized as a tumor suppressor in the early stage 16, 17. However, further studies on TAGLN revealed its strong potential as a tumor metastasis initiator 18-20. In colorectal cancer, the high expression of TAGLN is positively correlated with poor prognosis. Recent reports have also highlighted the important role of TAGLN in colorectal cancer. However, none of these studies explained the controversial TAGLN expression patterns. The exact expression pattern and molecular function of TAGLN in colorectal cancer is also unclear.

In this study, 471 patients with colorectal cancer and 41 normal colon tissues were analyzed to describe the differentially expressed genes (DEGs) of colorectal cancer and the functions of these DEGs. COMP was highly expressed in the colorectal cancer tissues, as observed through weighted gene co-expression network analysis (WGCNA) and Venn analysis. The high expression of COMP substantially affected the advanced clinicopathological features and poor prognosis of 597 patients. Moreover, COMP was found for the first time to interact with TAGLN to promote the migration and invasion of colorectal cancer cells. On the basis of the protein structure of the COMP/TAGLN complex, the specific inhibition of the COMP/TAGLN protein interaction by Chrysin from the in-house traditional Chinese medicine (TCM) database was determine through high-throughput screening technology 21. *In vivo* and *in vitro* experiments showed that Chrysin could significantly inhibit the proliferation and metastasis of colorectal cancer. The role of COMP and TAGLN in colorectal cancer was fully described by combining bioinformatics analysis and functional and mechanistic studies. This study may help improve the understanding of colorectal cancer metastasis and provide new targets for follow-up treatment.

## Materials and Methods

### Patient Samples

The expression of COMP and TAGLN in normal adenoma-adenocarcinoma sequences was evaluated by IHC staining from 45 human colon tissue specimens (15 non-neoplasia colon tissues, 15 adenoma tissues, and 15 colorectal cancer tissues). There were no significant differences in age or gender between the groups. These patients were excluded if they were suffering from other clinical diseases. All patients signed the informed consent, and ethical approval was obtained from the Ethics Committee of General Hospital, Tianjin Medical University, China 22.

### Cell culture and transfection

HCT116, HCT-8, and SW620 cells were purchased from KeyGEN BioTECH. Cells were cultured in RPMI 1640 or DMEM containing 10% FBS (Gibco, Life Technologies) and 1% penicillin and streptomycin (Hycult, Life Technologies). The cells were cultured in a cell incubator at 37 ℃ with 5% CO_2_, digested by trypsin, and subcultured every 2 days. Full-length COMP or mock (empty vector) or shCOMP was stably expressed in both cell lines by transfection with Lipofectamine 2000 (Invitrogen, USA) and selection with hygromycin or puromycin (Invitrogen, USA). COMP expression was verified by WB. Single clones with good COMP expression were chosen for further experiments.

### TCGA data downloading and DEG analysis

Human subjects were not involved in this study. The colorectal cancer data used were downloaded from the TCGA dataset, which contained 471 colorectal cancer and 41 normal colon tissue control samples. The DEGs were analyzed using the edgeR packages of Bioconductor. The cutoff values were set at FDR < 0.05 and |logFC| ≥ 2. A total of 969 DEGs, including 420 upregulated and 549 downregulated genes, were calculated.

### Gene set enrichment analysis (GSEA) analysis

GSEA software was used to input the gene expression matrix of colorectal cancer and normal control samples. All genes were sequenced to show the trend of gene expression between the two groups. The top and bottom of the sorted list of genes were viewed as the upregulated and downregulated DEGs, respectively. In GSEA results, ES indicated enrichment score, and the FDR q-value indicated q-value, which was the p value corrected by multiple hypothesis tests and represented the credibility of enrichment results. GSEA adopted the p- and q-values < 5% and < 25%, respectively, to filter the results.

### Cytoscape analysis (KEGG and GO)

The ClueGo plug-in in Cytoscape software was used to conduct pathway enrichment analysis of DEGs in colorectal cancer, and p < 0.05 was used as the threshold to set the standard for visual analysis of enrichment pathway results.

### WGCNA

A scale-free network topological analysis of mRNA expression data from colorectal cancer samples was performed via WGCNA. During the analysis, the data were collated and computed using the default standard parameters. Excel was used to sort the gene expression amount of patients with colorectal cancer and the age, lymphatic invasion, pathologic M, pathologic N, pathologic T, venous invasion, gender, tumor stage, status, and other information of each sample for follow-up analysis. In accordance with the expression of genes, the correlation of genes was determined; the genes with high expression correlation were clustered into a module. The correlation between the collected modules and the clinical data of patients was analyzed. The purpose of module clustering analysis was to integrate the acquired genes and further narrow the scope of key genes.

### CancerSEA analysis

CancerSEA (http://biocc.hrbmu.edu.cn/CancerSEA/) was used for the single-cell level comprehensive exploration database for cancer cell function. The CancerSEA database was used to analyze the COMP gene. The t-SEN distribution of COMP in all individual cells in colorectal cancer single-cell sequencing data was analyzed by functional correlation among different cell populations. The different colors represented the expression level of the input gene. Correlation analysis was conducted between COMP and the functions, angiogenesis, metastasis, and invasion of the EMT to further understand the COMP functions.

### UALCAN analysis

The UALCAN website (http://ualcan.path.uab.edu/cgi-bin/ualcan-res.pl) is the TCGA database data mining platform that provides the gene expression level and survival, correlation, and DNA promoter region methylation data analyses. This study directly investigated COMP expression in colorectal cancer and its relationship with prognosis.

### Human protein atlas analysis

The human protein atlas provides the protein level distribution of human protein in tissues and cells and uses IHC technology to check the distribution and expression of each protein in 48 normal human tissues, 20 tumor tissues, 47 cell lines, and 12 blood cells. The protein expression levels of TAGLN in normal colon tissues and colorectal cancer specimens were investigated, and the images were obtained from the human protein atlas online database.

### Immunopurification

Protein A/G agarose (50 μL, 50%; Pierce) was incubated with control or specific antibodies (1-2 μg) at 4 °C for 8 h. The HCT116 cell lysates were prepared on ice with 0.3% Nonidet P-40 lysis buffer under the action of the protease inhibitor mixture. The lysate was centrifuged at 12,000 rpm for 10 min at 4 °C. The precipitates were discarded, and the beads coupled with antibodies were incubated at 4 °C. After incubation for 12 h, the precipitates were washed with cold 0.1% Nonidet p-40 buffer five times. The precipitated protein was resuspended in 2×SDS-PAGE loading buffer, boiled at 99 °C for 10 min, and eluted from the beads. The boiled immune complex was subjected to SDS-PAGE and WB and then treated with COMP (1:1000) and TAGLN (1:1000).

### WB

Intracellular proteins were isolated using 10% or 15% SDS-PAGE gel and then transferred to polyvinylidene difluoride membranes. After 5% skim milk powder was sealed at room temperature for 1 h, the specific primary antibodies, including COMP (Abcam, ab231977, 1:1000), Vimentin (CST, 5741, 1:1000), E-cadherin (CST, 14472, 1:500), N-cadherin (Abcam, ab18203, 1:1000), Occludin (Affinity, AF0129, 1:1000), Twist1 (Sigma, 1:1000), Snail1 (Affinity, AF6032, 1:1000), MMP2 (Affinity, BF0005, 1:1000), and β-actin (Affinity, AF7018, 1:1000), were incubated overnight at 4 °C. The corresponding secondary antibodies were re-incubated, and the membranes were tested. Results were standardized using β-actin.

### Duolink

The Duolink experiment followed the steps described previously. In brief, a blocking solution was added to the samples for 30 min. After the blocking solution was removed, the samples were added with diluted primary antibodies (1:100). The primary antibodies were rinsed off for 3×5 min. The samples were added with PLA® PROBES, and two PLA probes were diluted and incubated for 1 h at 37 °C. The ligase was washed twice with Wash Dilute solution (1:40), added to the samples, and incubated for 30 min at 37 °C. The mixture was then washed in 1× Wash Buffer A for 2×2 min, and the amplification stock in water (1:5) was diluted. Polymerase was also diluted in the solution (1:80), added to the samples, incubated for 100 min at 37 °C, and washed with 1×Wash Buffer B for 2×10 min and 0.01×Wash Buffer B for 1 min. The samples were prepared for DAPI staining and confocal microscopy imaging.

### RNA interference

The Lipofectamine RNAi MAX (Invitrogen, USA) was used to transfect all siRNAs as recommended by the manufacturer. The final concentration of siRNA was 10 nM, and the cells were transfected for 72 or 96 h and collected for experiments.

### SEM

Cell slides were prepared in 24-well plates. After sticking to the wall, the cells were treated and fixed with 4% glutaraldehyde in PBS for 60 min. The slides were washed with PBS three times for 10 min, washed with 1% OsO_4_, and fixed for 60 min. The OsO_4_ solution was removed, and the cells were gently rinsed twice for 5 min. The cells were gradually dehydrated with gradient ethanol, and ethanol was replaced by gradient tert-butanol. Finally, the samples were subjected to vacuum drying, sprayed with gold, and imaged by SEM (LEO 1530 VP, Germany).

### Immunofluorescence assay

The cell slides were prepared in 24-well plates and treated as follows after cell adherence. The cells were washed three times with PBS for 5 min each time and fixed for 10 min by using precooled 3.7% formaldehyde. The sealed serum was diluted to 5% with 1×PBS (containing 0.1% Triton X-100 for cell wall penetration) at room temperature and incubated at room temperature for 30 min. The major antibodies used were TAGLN (diluted, 1:100), COMP (diluted, 1:100), E-cadherin (diluted, 1:100), and Vimentin (diluted, 1:100). After the incubation of cells with the primary antibody, the antibody was gently rinsed using PBS for three times and Alexa Fluor®488 (diluted, 1:150). Alexa Fluor®568 (diluted, 1:250) was used to mark the secondary antibody. The cells were observed and photographed by confocal microscopy (Nikon, Japan).

### Transwell experiment

In the invasion assay, the Transwell chamber was first coated with Matrigel (BD Biosciences), and cells of different treatments were added to the upper chamber of the Transwell. The lower chamber was treated with a medium containing 10% FBS. The cells were cultured at 37 °C for 24 h, washed three times with 1×PBS, fixed with 4% paraformaldehyde (precooled at 4 °C), and stained with crystal violet (KeyGEN BioTECH). The cells that passed were counted under a microscope (Nikon, Japan).

### Migration analysis

In the migration experiment, HCT116, HCT8, and SW620 cells with different treatments were uniformly scratched in the center of the cell culture plate. The cells after the scratch were photographed using a microscope every 24 h, and the gap size of the scratch was counted.

### Apoptosis assays

COMP- or mock-transfected cells (1:1 ratio) were cultured until 80% confluent in a six-well plate. The cells were treated with 15 μg/mL brefeldin A (Sigma-Aldrich, St Louis, MO, USA) or an equal volume of PBS for 48 h to induce apoptosis 5. Cell apoptosis induced by Chrysin (PS0183-0025, PUSH Bio-Technology, Chengdu, China) was examined by flow cytometry after 10 and 20 μM treatment. The cells were stained with an Annexin V-FITC apoptosis assay kit (Beyotime, Shanghai, China) and analyzed by flow cytometry.

### Xenograft tumor model

HCT116, HCT-8, and SW620 cells were infected *in vitro* with the COMP shRNA- or overexpressed plasmid. Subsequently, 2×10^6^ cells were injected into BALB/c nude mice (4-6 weeks old, Charles River, Beijing, China) bearing subcutaneous tumors. A total of 2×10^6^ HCT116 and HCT-8 cells or COMP knockdown cells were subcultured in BALB/c nude mice (4-6 weeks old) to study the inhibition of colorectal cancer due to Chrysin. After formation, the tumors were evenly divided by size. Experimental mice were treated with 20 mg/kg Chrysin every day. The control mice received the same amount of saline. The long and short diameters of the tumor were measured every 4 days by using a Vernier caliper. The tumor volume was calculated as follows: V = ab^2^/2 (a = tumor length, b = tumor width). After reaching the endpoint of the experiment, the mice were sacrificed, and the tumors were removed. The tumors were fixed using 4% paraformaldehyde, embedded in paraffin, and sectioned for subsequent detection.

A mouse model of liver metastasis was constructed as follows. Mice were anesthetized by intraperitoneal injection of 0.5% pentobarbital sodium (50 mg/kg, Sigma, USA). An incision of about 1 cm was made on the left side of the abdominal cavity. The spleen was gently isolated, and 2×10^6^ SW620 cells with stable overexpression and low COMP expression were suspended in PBS and injected into the spleen with a needle. The spleen was gently returned to the abdominal cavity, and the wound was sutured and disinfected. After splenic injection modeling, the experimental mice were treated with 20 mg/kg Chrysin every day to evaluate the Chrysin inhibitory effect on metastasis. After 5 weeks, the mice were sacrificed, and the liver was isolated to observe metastasis 23.

### IHC staining

The tumor sections were removed of paraffin by using gradient xylene and dehydrated with gradient ethanol. Hydrogen peroxide (3%) was used to block the endogenous levels of peroxidase. The protein was denatured in a microwave oven with citrate buffer brine (pH 6.0) to expose the antigen. At room temperature, normal goat serum was incubated for 30 min to seal the nonspecific binding. Anti-COMP (1:100), anti-E-cadherin (1:100), anti-Vimentin (1:100), anti-Snail1 (1:100), and anti-Twist1 (1:500) were incubated overnight at 4 °C. Diaminobenzidine and hematoxylin were used as color developing and redyeing agents, respectively. The expression levels of COMP, E-cadherin, Vimentin, Snail1, and Twist1 were independently evaluated by two researchers.

### Protein-protein docking and molecular screening

The protein crystal structure of COMP and TAGLN was downloaded from the PDB (http://www.rcsb.org) protein database. Hydrogenation, steric hindrance optimization, and hydrogen bond optimization were carried out on the initial structure to eliminate stereoscopic conflict. Protein docking was performed using HEX software. COMP was selected as receptor, and the TAGLN structure was selected as the ligand. The docking parameters were selected based on the protein surface structure and surface potential. On the basis of the COMP/TAGLN complex structure obtained by docking, the protein interaction interface was selected as the active pocket for high-throughput screening of the TCM database. The XYZ size of the docking grid box was set to 60 × 60 × 60. Ligprep was used to generate the three-dimensional structure of the TCM monomer, and the OPLS-2005 force field was used to minimize the energy of the structure.

### Biacore analysis

Chrysin and COMP solutions were prepared, and channel 4 of the CM5 chip was activated. MNHS (50 mM) and MEDC (200 mM) solutions were mixed in equal proportions. The mixture was then injected at a rate of 10 μL/min, followed by COMP at 10 μL/min. The activation of the conjugated protein steps was repeated until the desired signal strength was reached. The remaining active groups were injected into the chip channel at a rate of 10 µM/min to seal it. The buffer used by the system was 1×PBS. Small molecules of Chrysin (0, 1.6, 3.125, 6.25, 12.5, and 25 μM) were injected at a rate of 30 μL/min for 180 s. In the dissociation phase, the running buffer was injected at a rate of 30 μL/min for 300 s. All signals were corrected by channel 1 as the control channel. The data were analyzed and processed using BIAevaluation Software (Biacore, GE Healthcare).

### Statistical analysis

Statistical analyses were performed using GraphPad Prism (GraphPad Software, Inc., USA). Data from biological triplicate experiments were presented with an error bar as the mean ± standard deviation. Two-tailed unpaired Student's t-test was used to compare two groups of data, whereas one-way ANOVA was used to compare multiple groups of data. According to the statistical correlation requirements, Pearson correlation coefficient was used to calculate the co-expression correlation of the two proteins. A survival curve was generated by the Kaplan-Meier method and assessed by a log-rank test. Differences were considered significant when *p < 0.05, **p < 0.01, or ***p < 0.001.

## Results

### Analysis of DEGs, functions, and pathways in colorectal cancer

**Figure 1:**
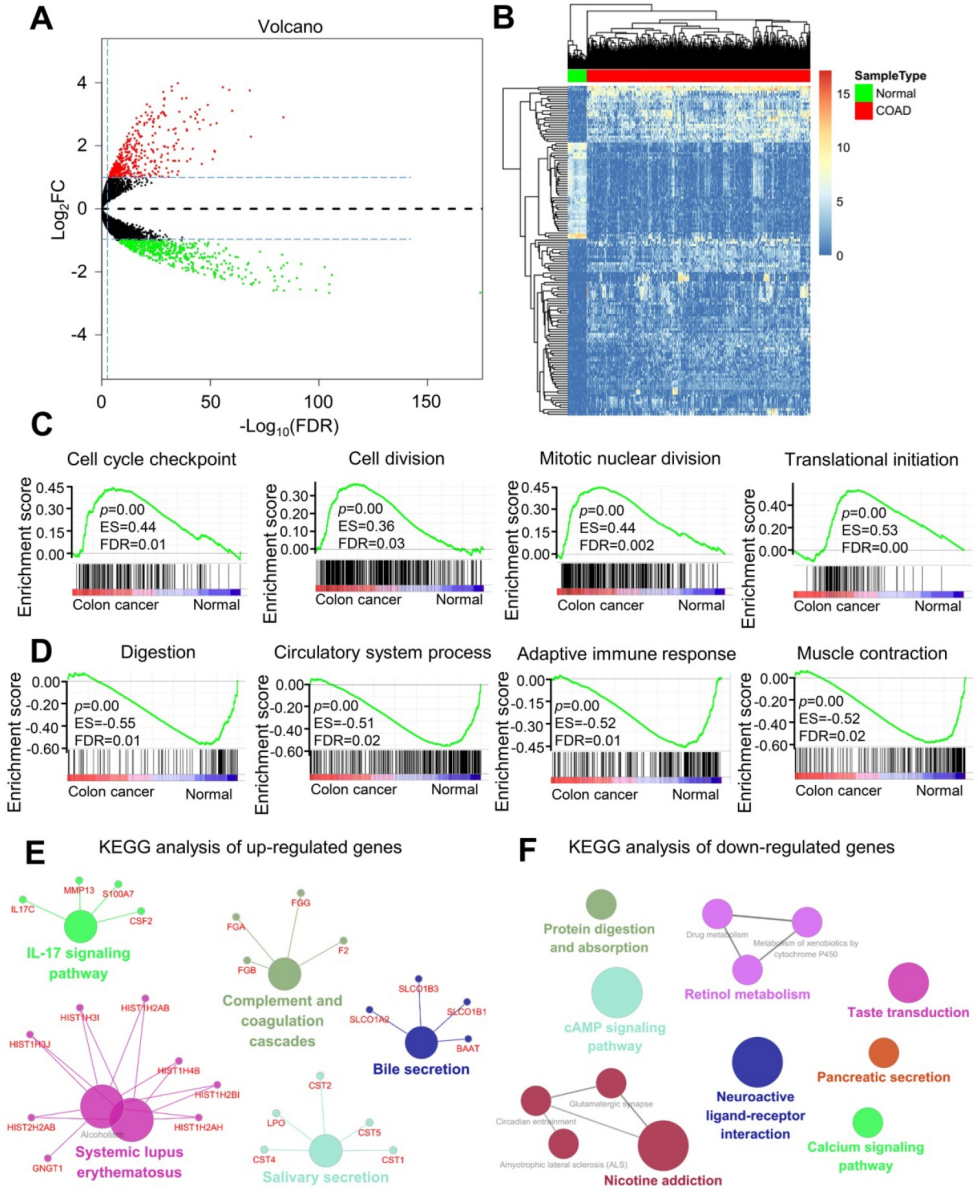
** Analysis of differentially expressed genes (DEGs) and differential function and pathway enrichment in colorectal cancer and normal samples.** (**A**) Volcano map of DEGs between colorectal cancer and normal colon tissues. The threshold was set as twice the difference, and the FDR value was less than 0.05. The red and green dots represent upregulated and downregulated genes, respectively, and the black dots represent non-DEGs. (**B**) Heatmap of the DEGs according to the value of |logFC|. The green and red modules represent the normal and colorectal cancer samples, respectively. (**C and D**) Gene set enrichment analysis (GSEA) of genes in the colon and normal tissues. (**E and F**) KEGG enrichment analysis of (E) upregulated and (F) downregulated genes.

The DEGs in colorectal cancer were identified, and enrichment analysis was conducted. Colorectal cancer and normal control colon tissues were pretreated using the limma package. With p < 0.05 and |logFC| > 1 as the threshold, 969 DEGs were identified. Among these DEGs, 420 upregulated (Table S1) and 549 downregulated (Table S2) genes were found in colorectal cancer tissues (Figure 1A). DEGs are shown in the volcano diagram, and the first 100 DEGs shown based on the value of |logFC| are also revealed in the heat diagram (Figure 1B). Cytoscape software was used to compare the biological process of gene cluster enrichment, and the cutoff value was p < 0.05. In Gene Ontology (GO) analysis, significantly enriched upregulated genes included the cell cycle checkpoint, cell division, mitotic nuclear division, and translation initiation (Figure 1C). The downregulated genes were significantly enriched in digestion, circulatory system, adaptive immune response, and muscle contraction (Figure 1E). In the Kyoto Encyclopedia of Genes and Genomes (KEGG) analysis, the upregulated genes were mainly enriched in the complement and coagulation cascades and the IL-17 signaling pathway. The downregulated genes were mainly concentrated in protein digestion and absorption, calcium signaling pathway, cAMP signaling pathway, and neuroactive ligand-receptor interaction (Figure 1F). Significant enrichment of functions and pathways may help us further investigate the role of DEGs in colorectal cancer.

### WGCNA analysis of colorectal cancer genes

WGCNA was conducted to describe the correlation between the gene expression levels and clinical information of patients with colorectal cancer and further screen key genes in colorectal cancer. WGCNA is a systematic biological approach that describes the pattern of genetic associations among patients with different colorectal cancers. It can identify candidate biomarker genes or therapeutic targets based on the association of gene sets and the association between gene sets and phenotypes. WGCNA uses information from thousands of the most variable genes in colorectal cancer to identify the genes of interest that are significantly correlated with the clinical phenotype of patients with colorectal cancer. Finally, the key genes of colorectal cancer can be enriched and selected.

**Figure 2:**
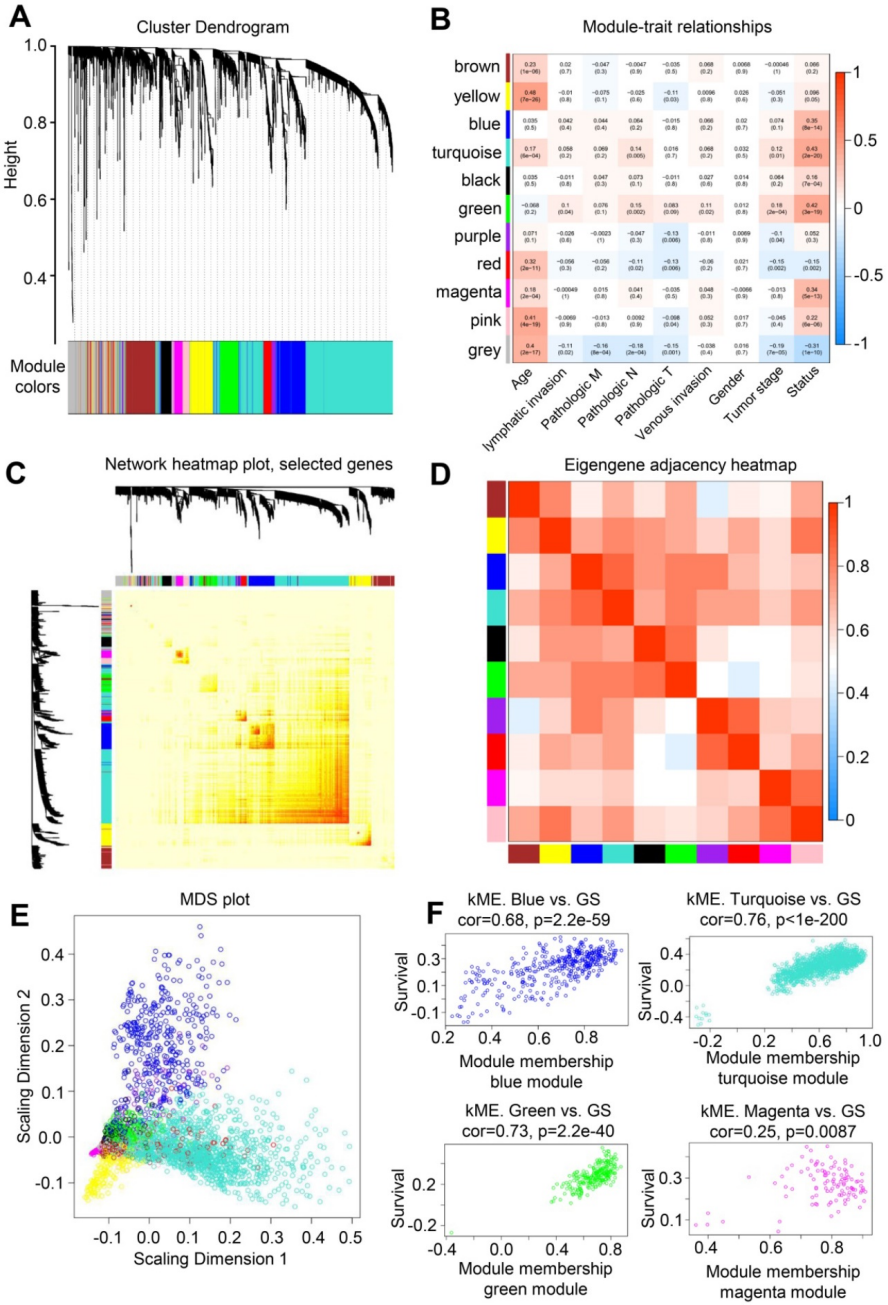
** WGCNA analysis of colorectal cancer and normal samples.** (**A**) Genes were assigned to one of the 11 modules, including the Gray module. The sequence diagram is shown at the top of the image. The bottom of the figure shows the different color gene modules and number of bases they contain. (**B**) Correlation analysis between module and sample clinical information. The number on each cell is the correlation coefficient between each module gene and clinical information, and the number below is the corresponding p value. Among these modules, the Turquoise module is the most relevant to tumor characteristics. (**C**) Results of heatmap analysis of 1000 randomly selected genes. The strength in red indicates the strength of the linear relationship between the module pairs. (**D**) Eigengene adjacency heatmap of each module, representing the correlation between the modules. (**E**) Multidimensional scaling (MDS) plot of each module. MDS uses the similarity between the paired samples to construct appropriate low-dimensional space to visualize the data distribution of each module. (**F**) A scatter plot of GS for colorectal cancer versus the MM in the Blue, Turquoise, Green, and Magenta modules. Intramodular analysis of genes found in the Turquoise module, which contains genes that are highly correlated with colorectal cancer (p < 1e-200, correlation = 0.76).

A highly synergistic set of genes was integrated to divide colorectal cancer expression genes into 11 modules through WGCNA (Figure 2A). The correlation between these 11 gene set modules and the clinical information of patients with colorectal cancer was further determined. The gene in the Turquoise block had the highest correlation with the survival status of patients (Figure 2B), and 1000 randomly selected genes were used for network heatmap analysis (Figure 2C). The Eigengene adjacency heatmap was based on the gene expression quantity between clustering of correlation among various modules (Figure 2D). In addition, multidimensional scaling analysis was performed on the genes in 11 modules (Figure 2E). The gene in the Turquoise module was highly correlated with the survival status of patients with colorectal cancer, with a correlation coefficient of 0.76 (Figure 2F). Thus, the genes in this module could act as hub genes for colorectal cancer.

### COMP expression is related to the malignancy degree of colorectal cancer

**Figure 3:**
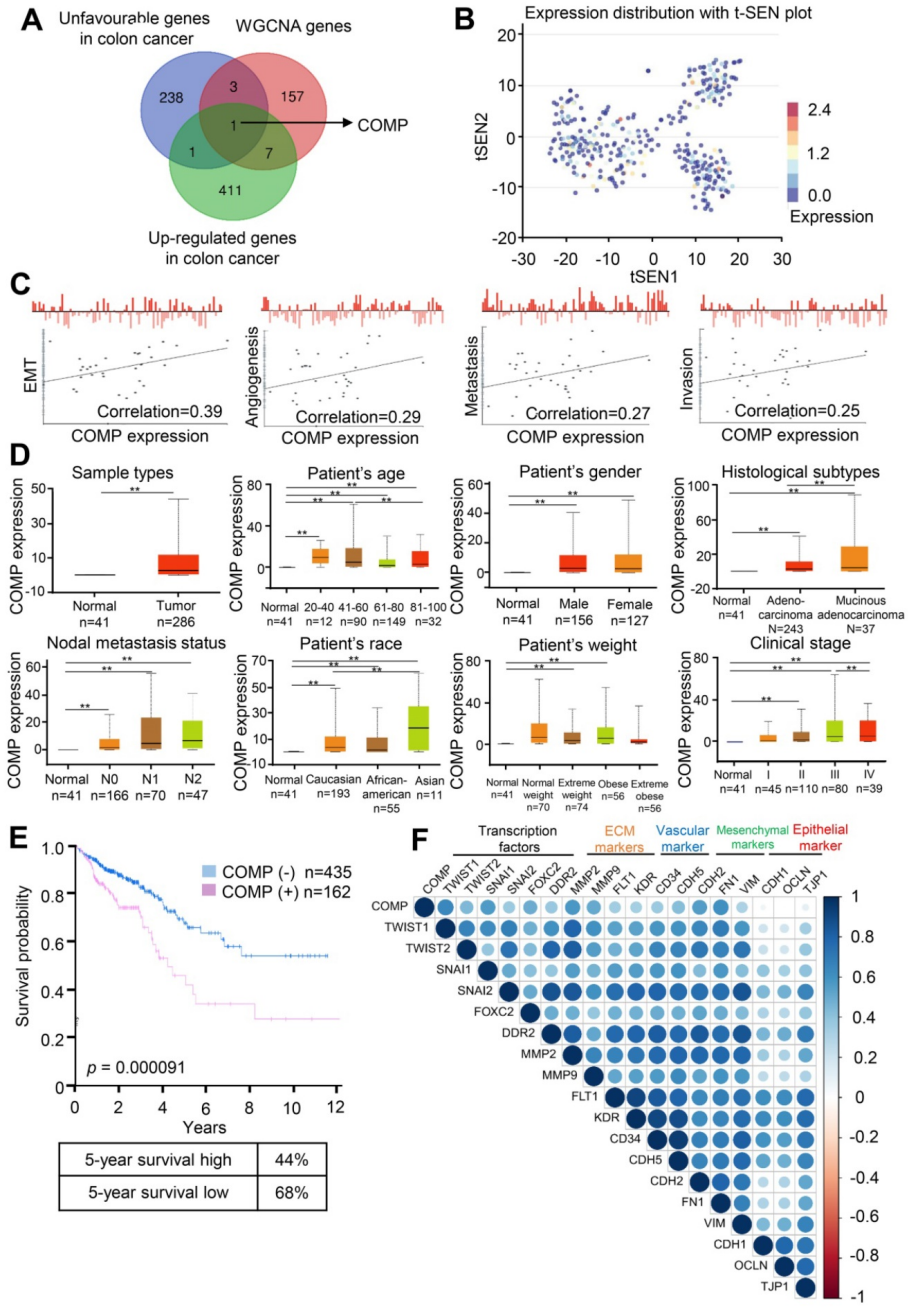
** COMP is upregulated in colorectal cancer and associated with poor prognosis.** (**A**) Venn analysis of WGCNA important genes, genes with poor prognosis in colorectal cancer, upregulated gene expression in colorectal cancer, and tumor vascular-related genes. (**B**) Expression distribution of COMP in colorectal cancer cells in the CancerSEA database. Every point represents a single cell, and the color of the point represents the expression level of COMP. (**C**) Functional state analysis of COMP in colorectal cancer cells. (**D**) Expression analysis of COMP in normal colon tissues and colorectal cancer tissues. Box plot shows the relative expression of COMP in normal and colorectal cancer samples (UALCAN). Results revealed that PAI-1 was highly expressed in patients with colorectal cancer, and COMP expression was positively correlated with lymph node metastasis and pathological staging in patients with colorectal cancer. (**E**) Survival analysis of COMP in patients with colorectal cancer. The red and blue lines represent patients with high and low expression levels of COMP, respectively. The X axis indicates overall survival time (years), and the Y axis indicates the survival rate. A Kaplan-Meier test was performed. (**F**) EMT genes correlated with COMP expression.

The correlation between the gene set and the clinical phenotype of patients with colorectal cancer was determined through WGCNA, but the module itself still contained a large number of genes. The most important genes should be further searched. To extract valuable clues from these data, key genes were discovered from the poor prognosis of colorectal cancer gene sets (Table S3), WGCNA genes (Table S4), and upregulated expression genes in colorectal cancer. Finally, COMP was identified (Figure 3A). Figure 2B shows the t-SEN diagram of COMP in all individual cells in the single-cell sequencing data set of colorectal cancer, where the color depth represents the expression of COMP. The correlation analysis between the expression level of COMP and cell function showed that the expression of COMP was positively correlated with EMT, angiogenesis, metastasis, and invasion of colorectal cancer cells (Figure 3C). COMP expression in colorectal cancer tissues was significantly higher than that in normal tissues (p < 0.05). Patients with colorectal cancer with high COMP expression also had higher metastasis and clinical staging than those with low COMP expression (Figure 3D). Patients with high COMP expression had significantly shorter overall survival than those with low COMP expression (Figure 3E). The results of expression analysis showed that COMP was co-expressed with Twist1, Twist2, SNAI1, SNAI2, and FOXC2. COMP was positively correlated with the expression of extracellular matrix-related (DDR2, MMP2, and MMP9) and vascular-related (FLT1, KDR, CD34, and CDH5) genes. COMP was co-expressed with mesenchymal markers (CDH2, FN1, and VIM) and negatively correlated with epithelial markers (CDH1, OCLN, and TJP1; Figure 3F).

### COMP promotes EMT in colorectal cancer

**Figure 4:**
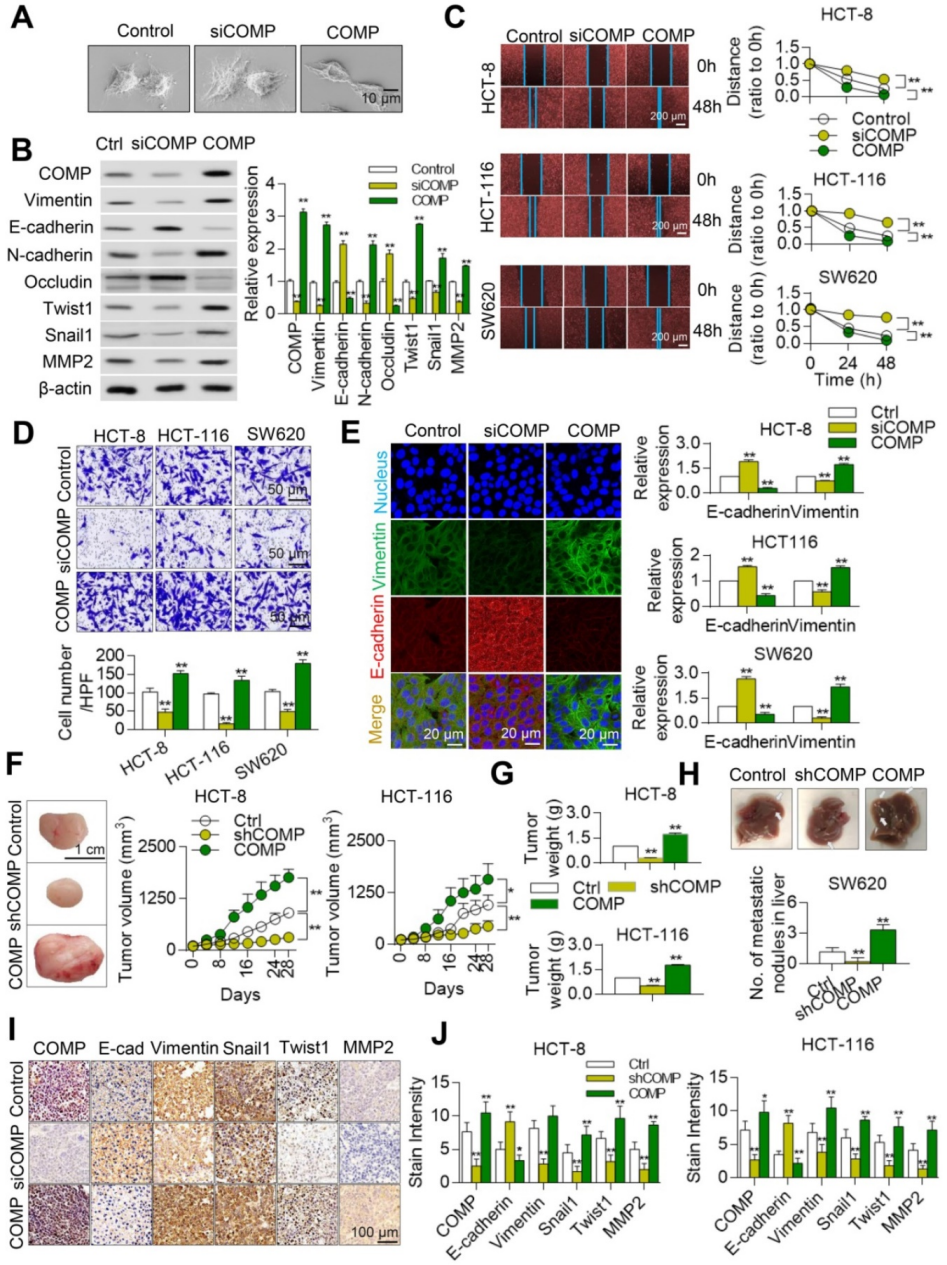
** COMP promotes EMT in colorectal cancer.** (**A**) Cell phenotypic changes in cells treated with COMP siRNA and COMP overexpression. (B) WB analysis of COMP and EMT-related markers in HCT116 cells under COMP knockdown or overexpression. (**C**) Cell wound scratch assay of HCT116, HCT-8, and SW620 cells treated with COMP siRNA or COMP overexpression vectors. (**D**) Transwell assay of HCT116, HCT-8, and SW620 cells treated with COMP siRNA or COMP overexpression vectors. (**E**) Immunofluorescence assay of HCT116, HCT-8, and SW620 cells treated with COMP siRNA or COMP overexpression vectors. Image J software was used to analyze the relative intensity of E-cadherin and Vimentin. (**F**) HCT116 and HCT-8 cells with knocked down or overexpressed COMP were transplanted on nude mice. Tumor volumes were measured every 4 days. (**G**) Tumor weight in the control, COMP knockdown, and COMP overexpression groups. (**H**) The *in situ* spleen model of the colorectal cancer cell line SW620 showed that overexpression of COMP promoted liver metastasis of colorectal cancer, while downregulation of COMP inhibited liver metastasis of colorectal cancer. (**I**) IHC staining to identify EMT biomarkers and COMP-related proteins in the control, COMP knockdown, and COMP overexpression groups. COMP knockdown displayed strong E-cadherin staining and reduced Vimentin, Snail1, Twist1, and MMP-2 staining. The expression levels of other proteins were identical to those observed in WB experiments. (**J**) Staining indices of COMP, E-cadherin, Vimentin, Snail1, Twist1, and MMP2. The error bars in all graphs represented SD, and each experiment was repeated three times. * and ** stand for P<0.05 and P<0.01, respectively.

The effect of COMP in the EMT of colorectal cancer cells based on the biological function analysis of COMP was studied to understand the biological significance of COMP. Scanning electron microscopy (SEM) results showed that COMP knockdown promoted epithelial changes in colorectal cancer cells, whereas COMP overexpression promoted mesenchymal phenotypic changes in colorectal cancer cells (Figure 4A).

Western blot (WB) results further confirmed that the expression levels of epithelial markers (E-cadherin and occludin) were upregulated after the knockdown of COMP by colorectal cancer cells. In addition, the expression levels of mesenchymal markers (Vimentin and N-cadherin) and those of Twist1, Snail1, and MMP2 decreased in the siCOMP group. The overexpression of related markers of COMP and EMT led to opposite findings (Figure 4B). The invasion and migration abilities of cells were evaluated by Transwell and wound healing experiments, respectively. COMP knockdown inhibited the invasion and migration of HCT116, HCT-8, and SW620 cells, whereas COMP overexpression promoted the invasion and migration of cells (Figures 4C and 4D). The apoptosis experimental results showed that overexpression of COMP inhibited the apoptosis induced by Brefeldin A of SW620, while COMP knockdown promoted the apoptosis of SW620 (Figure S1). Consistent with the WB results, the immunofluorescence results revealed that COMP knockdown increased and decreased the fluorescence intensities of the epithelial marker E-cadherin and Vimentin, respectively, and COMP overexpression presented opposite results (Figure 4E).

Stable COMP-deficient and COMP-overexpressed HCT116 and HCT8 cells were constructed and transplanted into the subcutaneous region of mice to establish a xenograft model and investigate the role of COMP in promoting malignant progression *in vivo*. Compared with the control group, the COMP-deficient nude mice had slow tumor growth and small tumor volume, and the COMP-overexpressed nude mice exhibited fast tumor growth and large tumor volume (Figure 4F). Tumor weight decreased in the COMP knockdown group but increased in the COMP overexpression group (Figure 4G). The in-situ spleen injection experiment was adopted to determine the effect of COMP on the metastasis of CRC cells *in vivo*. In contrast to the SW620 control group, overexpression of COMP significantly increased the metastasis of SW620 cells to the liver. Knockdown of COMP inhibited liver metastasis in colorectal cancer (Figure 4H).

The expression levels of COMP, E-cadherin, Vimentin, Twist1, Snail1, and MMP2 in tumors were detected by immunohistochemistry (IHC) staining (Figure 4I). The results showed decreased expression levels of COMP, Vimentin, Twist1, Snail1, and MMP2 and increased expression of E-cadherin in the COMP-deficient group but increased expression levels of Vimentin, Twist1, Snail1, and MMP2 and decreased expression of E-cadherin in the COMP-overexpressed group. Thus, COMP increased the growth of colorectal cancer (Figure 4J).

### COMP is physically associated with TAGLN

**Figure 5:**
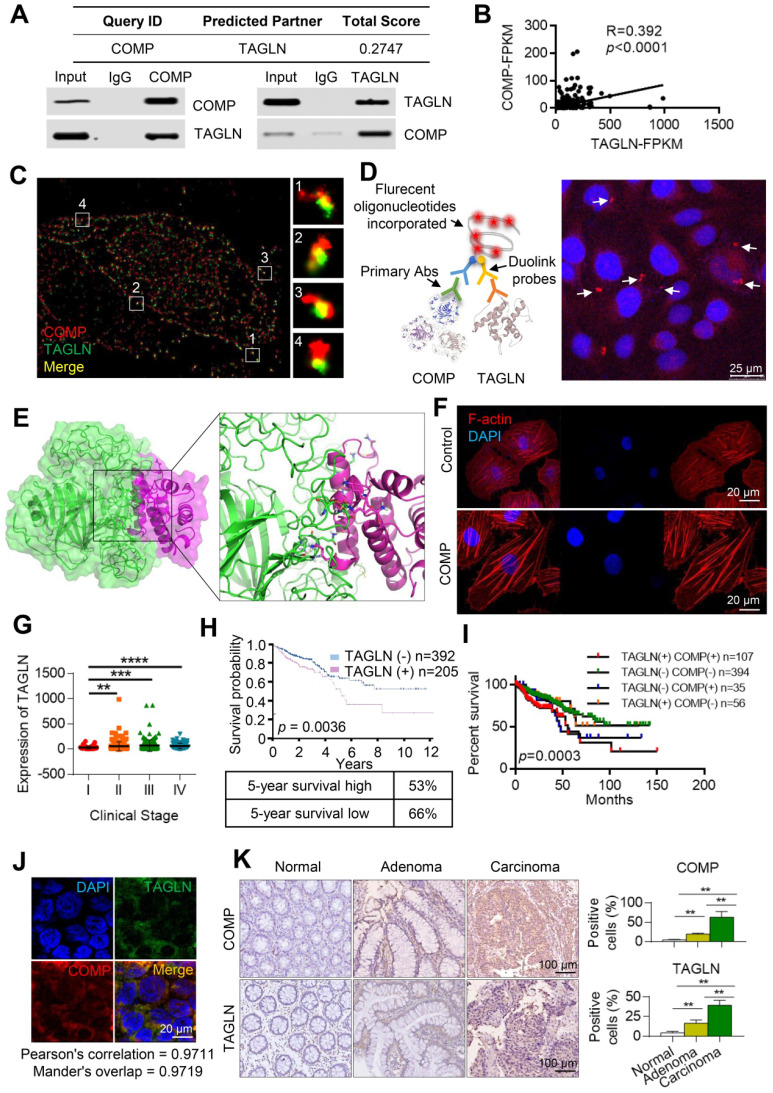
** COMP is physically associated with TAGLN.** (**A**) The interaction between COMP and TAGLN as predicted by the FpClass website and verified through co-immunoprecipitation experiments. (**B**) Statistical analysis of the correlation analysis between the expression of COMP and TAGLN in colorectal cancer samples of TCGA data. (**C**) Ultrahigh resolution microscopy verified the colocalization of COMP and TAGLN in colorectal cancer cells. Red and green fluorescence represent COMP and TAGLN, respectively. (**D**) The Duolink experiment verified the interaction between COMP and TAGLN. The red fluorescence represents the fluorescence point where COMP interacts with TAGLN. (**E**) Protein-protein docking of COMP and TAGLN and the interaction interface of amino acid in the binding site. (**F**) F-actin staining of colorectal cancer cells. The overexpression of COMP promoted F-actin aggregation. (**G**) Analysis of the expression levels of TAGLN in TCGA COAD samples based on clinical stages. (**H**) Survival analysis and 5-year survival rate of TAGLN in colorectal cancer samples. (**I**) Kaplan-Meier curve showing the survival rate of colorectal cancer samples classified by COMP or TAGLN co-expression. (**J**) The co-expression of COMP and TAGLN in colorectal cancer tissues was detected. Green fluorescence represents TAGLN, red fluorescence represents COMP, and orange fluorescence represents Merge results. (**K**) COMP and TAGLN expression for the normal-adenoma-adenocarcinoma sequence (n=15). The error bars in all graphs represented SD, and each experiment was repeated three times. *, **, and *** stand for P<0.05, P<0.01, and P<0.001, respectively.

The COMP interaction was analyzed to fully understand the function of COMP. COMP was found to be associated with several protein interactions through the FpClass website (http://dcv.uhnres.utoronto.ca/FPCLASS/ppis/). TAGLN, an actin-binding protein, was also found in the COMP-containing protein complex. The interaction between COMP and TAGLN was further verified by co-immunoprecipitation. COMP efficiently co-immunoprecipitated with TAGLN, and TAGLN was efficiently co-immunoprecipitated with COMP (Figure 5A). The correlation between the expression of COMP and TAGLN was analyzed by using the sequencing data from patients with colorectal cancer in The Cancer Genome Atlas (TCGA) database. A significant positive correlation was found between the expression levels of COMP and TAGLN (p < 0.001, R = 0.392; Figure 5B).

Immunofluorescence experiments were performed on COMP and TAGLN in HCT116 and HCT-8 cells by using the N-STORM technique to further analyze the interaction between COMP and TAGLN. COMP and TAGLN were found to be colocated (Figure 5C). The interaction between COMP and TAGLN was verified by the Duolink experiment (Figure 5D). Immunofluorescence staining was used to accurately analyze the multidirectional colocalization of the two proteins. The interfacial interaction of amino acids between COMP and TAGLN was analyzed and demonstrated through a protein-protein molecular docking experiment (Figure 5E). As an actin-binding protein, TAGLN promoted the aggregation of G-actin to F-actin. The cell function experiments showed that COMP overexpression increased the F-actin content in cells possibly through the enhanced interaction with TAGLN (Figure 5F).

Colorectal cancer transcriptome data analysis in the TCGA database (http://ualcan.path.uab.edu/index.html) showed that TAGLN expression was positively correlated with clinical stage (Figure 5G). Survival analysis revealed that the high expression of TAGLN suggested the poor prognosis of colorectal cancer (Figure 5H). The combined survival analysis of COMP and TAGLN also displayed that patients with high co-expression of COMP and TAGLN had the worst prognosis compared with the other patients (Figure 5I). COMP and TAGLN showed significant co-localization at the colorectal cancer tissue levels ((Pearson's correlation = 0.9711; Figure 5J). The expression of COMP and TAGLN significantly increased during the colorectal normal-adenoma-adenocarcinoma sequence (all p< 0.05), and patients with high expression of COMP and TAGLN had high clinical staging (Figures 5K and S2).

These results suggested that the COMP/TAGLN axis promoted the malignant progression of colorectal cancer. Therefore, COMP/TAGLN may be a potential target for colorectal cancer therapy.

### Chrysin targets the COMP/TAGLN complex

**Figure 6:**
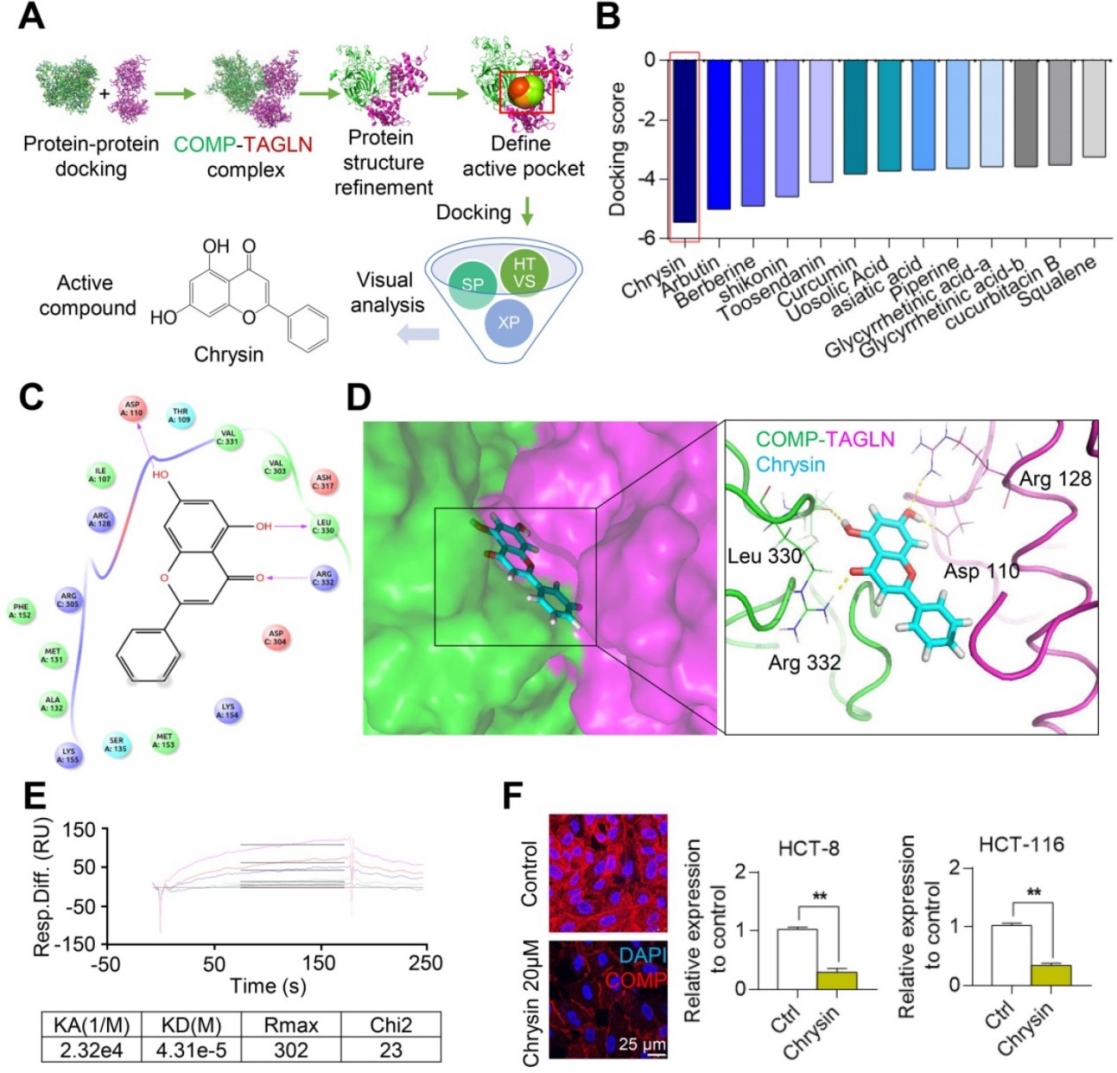
** Chrysin targets and combines with the interactive interface of the COMP/TAGLN complex.** (**A**) Screening of the Chinese medicine monomer library based on the COMP/TAGLN interaction interface. (**B**) Molecular docking results of the COMP/TAGLN complex with the lead compounds chosen from TCM. (**C**) Binding model of the Chrysin-targeted COMP/TAGLN complex. (**D**) Chrysin and COMP/TAGLN combined amino acids, and their interaction modes were demonstrated by a three-dimensional structure. (**E**) Biacore analysis of the interaction between Chrysin and COMP. (**F**) HCT116 and HCT-8 cells treated with Chrysin were studied by immunofluorescence analysis. The error bars in all graphs represented SD, and each experiment was repeated three times. * and ** stand for P<0.05 and P<0.01, respectively.

On the basis of the structure of the COMP/TAGLN complex, the binding site of the complex was set as an active pocket, and the lead compound with the highest score for docking with the COMP/TAGLN complex was selected from the TCM database by using high-throughput molecular docking virtual screening (Figures 6A and 6B). The molecular docking simulation showed the binding mode of Chrysin and the COMP/TAGLN complex. The docking results showed that four hydrogen bond interactions were formed between Chrysin and Asp110, Leu330, and Arg332 in the COMP/TAGLN complex. The hydrophobic interactions were formed with Val303, Val331, and Leu330, and π-π PI conjugation was formed with Lys154, Met153, and Ser135 (Figures 6C and 6D). The interaction between Chrysin and COMP was further verified by the Biacore experiment (Figure 6E). HCT116 and HCT-8 cells were treated with Chrysin, and immunofluorescence analyses were performed to investigate the effect of Chrysin on COMP. The immunofluorescence experiments demonstrated that Chrysin could inhibit the expression of COMP (Figure 6F).

### Chrysin inhibits the EMT of colorectal cancer

**Figure 7:**
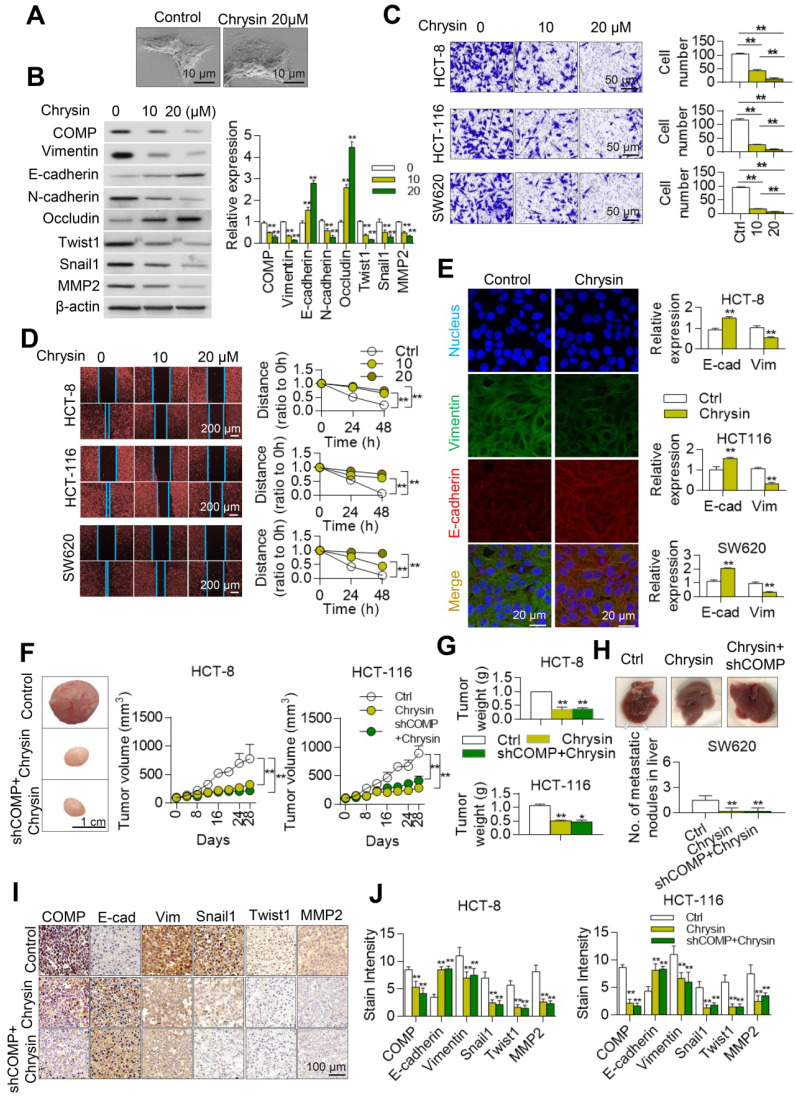
** Chrysin inhibits the EMT of colorectal cancer.** (**A**) Cell phenotypic changes in HCT116 cells treated with 20 µM Chrysin. (**B**) WB analysis of COMP and EMT-related markers in HCT116 cells treated with different concentrations of Chrysin. (**C**) Matrigel invasion assay of HCT116, HCT-8, and SW620 cells treated with different concentrations of Chrysin. (**D**) Cell wound scratch assay of HCT116, HCT-8, and SW620 cells treated with different concentrations of Chrysin. (**E**) E-cadherin and Vimentin immunofluorescence assays after Chrysin treatment of colorectal cancer cells for 24 h. (**F**) HCT116 and HCT-8 cells were injected to nude mice subcutaneously and treated with Chrysin. Tumor volumes were measured every 4 days. (**G**) Tumor weight in the control, Chrysin, and Chrysin+shCOMP groups. (**H**) The *in situ* spleen model of the colorectal cancer cell line SW620 showed that Chrysin inhibited liver metastasis of colorectal cancer. (**I**) Immunohistochemical staining to identify EMT biomarkers and COMP-related proteins in the control, Chrysin, and Chrysin+shCOMP groups. (**J**) Staining indices of COMP, E-cadherin, Vimentin, Snail1, Twist1, and MMP2 in the control, Chrysin, and Chrysin+shCOMP groups. The error bars in all graphs represented SD, and each experiment was repeated three times. * and ** stand for P<0.05 and P<0.01, respectively.

A biological laboratory study of Chrysin was conducted *in vivo* and *in vitro* to further study the inhibitory effect of Chrysin on colorectal cancer through the COMP/TAGLN complex. Chrysin could inhibit the mesenchymal transformation of colorectal cancer cells, and Chrysin treatment transformed the cell phenotype into the epithelial phenotype, showing decreased pseudopod and evident adherence to the wall (Figure 7A). Chrysin inhibited the expression of mesenchymal markers and upregulated the expression of epithelial markers (Figure 7B). Chrysin also inhibited the invasion and migration of HCT116, HCT8, and SW620 cells in a dose-dependent manner (Figures 7C and 7D). Chrysin induced apoptosis of SW620 cells in a dose-dependent manner (Figure S3). The immunofluorescence assay further revealed that Chrysin treatment downregulated the expression level of mesenchymal markers (Vimentin) and upregulated the expression level of the epithelial markers (E-cadherin) in HCT116, HCT-8, and SW620 cells (Figure 7E).

**Figure 8:**
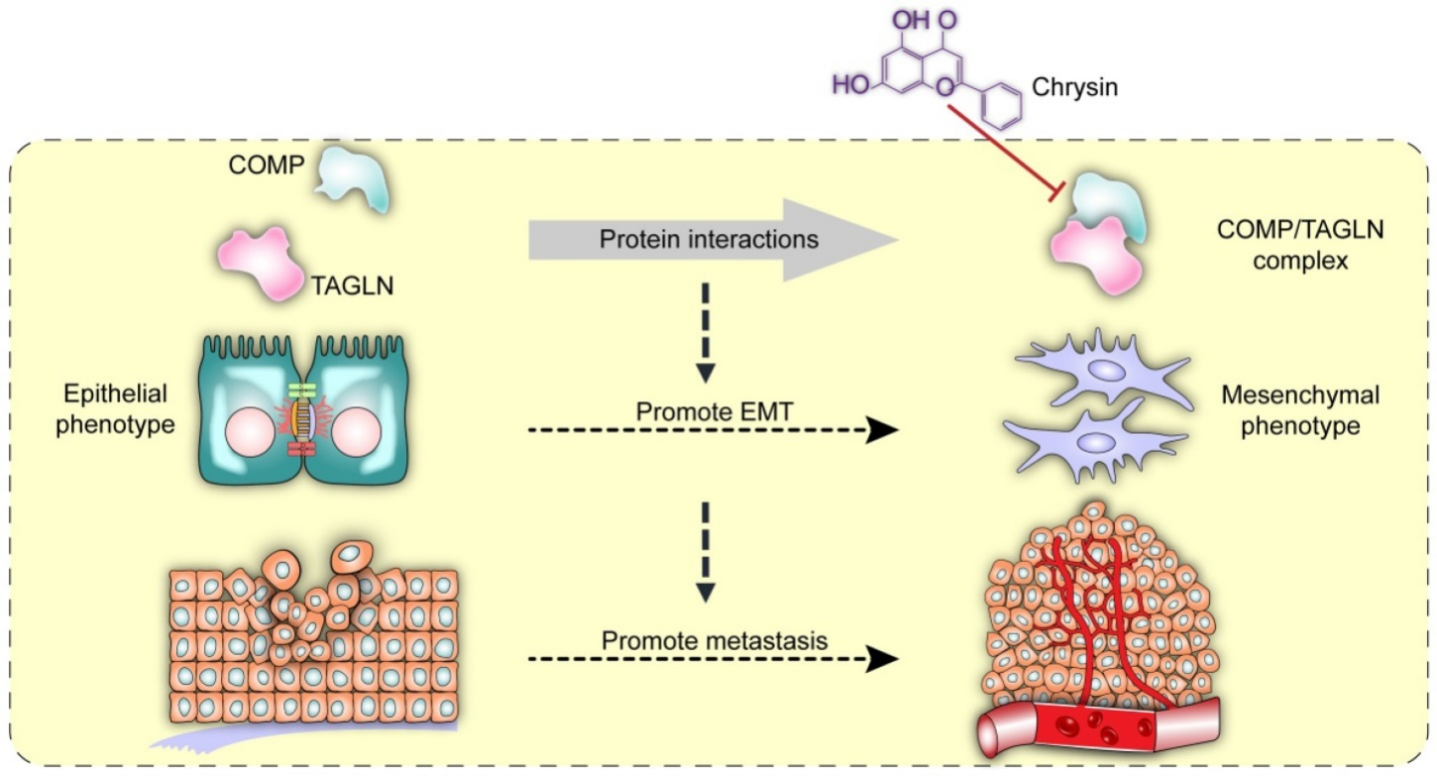
** Interaction between COMP and TAGLN promoted EMT in colorectal cancer.** The COMP/TAGLN complex regulates tumor cytoskeleton and promotes tumor metastasis, while Chrysin can inhibit the COMP/TAGLN complex and malignant tumor evolution. CRC, colorectal cancer. COMP, Cartilage oligomeric matrix protein. TAGLN, Transgelin. EMT, epithelial-mesenchymal transition.

Chrysin therapy and/or combined COMP knockdown (Figure 7F) were applied to the xenograft tumor models to verify the inhibitory effect of Chrysin on colorectal cancer *in vivo*. The experimental results showed that the Chrysin treatment group demonstrated decreased tumor volume and tumor weight (Figure 7G) and was not significantly different from the shCOMP+Chrysin treatment group. The effect of Chrysin was determined by spleen injection of SW620 cells. Chrysin significantly inhibited the hepatic metastasis of SW620 compared with the control group and showed no significant difference from the shCOMP+Chrysin treatment group (Figure 7H). In addition, the expression levels of COMP, E-cadherin, Vimentin, Snail1, Twist1, and MMP2 were measured by IHC. The results showed decreased expression levels of COMP, Vimentin, Snail1, Twist1, and MMP2 and increased expression of E-cadherin after Chrysin treatment. The Chrysin and shCOMP+Chrysin treatment groups showed no significant difference (Figures 7I and 7J). Chrysin inhibited the EMT in colorectal cancer, and this mechanism was achieved by targeting the COMP/TAGLN complex (Figure 8).

## Discussion

Approximately 10% of the world's annual cancer and cancer-related deaths are due to colorectal cancer, and the morbidity and mortality of men are approximately 25% higher than those of women 24. To date, colorectal cancer is the fourth most deadly cancer worldwide, killing approximately 900,000 people every year. The diagnosis and treatment of colorectal cancer are important because symptoms appear only in the advanced stages of the disease. EMT is an important biological process for malignant tumor cells to migrate and invade 25. Elucidating the molecular mechanism of EMT regulation in malignant tumor cells and clarifying its role in the occurrence, development, and metastasis of malignant tumors are crucial. Exploring diagnostic methods based on EMT key molecules and the treatment of key molecules targeting EMT are key scientific issues in research on tumor metastasis mechanisms.

In this study, FpClass prediction and protein immunoprecipitation experiments were performed, and results showed that COMP interacted with TAGLN. COMP can induce the expression of apoptosis inhibitors and prevent apoptosis 5-8, 26. This study found that COMP was involved in the malignant progression of colorectal cancer, and its mechanism may be related to the expression of apoptosis inhibitors induced by COMP and the inhibition of apoptosis in colorectal cancer cells. In addition, COMP binds directly to thrombin, inhibits thrombin activity, and prevents physiological hemostasis and thrombosis 27. This function of COMP may also increase the risk of colorectal cancer metastasis. Stromal cells inhibit tumor metastasis through the ECM barrier, which tumor cells must break through to metastasize 5. Studies have shown that COMP can increase the ability of tumor cells to degrade the surrounding ECM stroma and enhance EMT by upregulating the expression of MMP9, thereby promoting tumor metastasis 5. COMP was also reported to enhance the invasion ability of prostate cancer cells (DU145 cells) by binding to integrin 28.

TAGLN, a member of the calmodulin family, acts as an actin-binding protein that regulates cytoskeletal remodeling by promoting actin aggregation 15. The cellular immunofluorescence experiments and SEM showed that COMP could interact with TAGLN to regulate cell remodeling and promote EMT in tumor cells. TAGLN was recently reported to promote bladder cancer metastasis by inducing invadopodia formation and EMT 20. Therefore, studies on the interaction between COMP and TAGLN and their biological functions are important (Figure 8).

Protein-protein interaction (PPI) is associated with many biological effects. Thus, small molecules that specifically interfere with protein-protein recognition are becoming increasingly important in future antitumor drug development. However, some characteristics and properties of the protein complex interaction interface, such as large interaction interface and flat binding interface, introduce difficulties to PPI and inhibition research. In this study, COMP and TAGLN were not kinases and did not have traditional drug binding pockets. However, their PPI interface could be used as a good active site. Some properties and characteristics of the interaction interface between COMP and TAGLN were analyzed and summarized, and Chrysin from the TCM monomer database was screened to obtain the highest docking scores. Finally, the combination of COMP and Chrysin and the inhibitory effect of Chrysin on colorectal cancer were demonstrated through *in vivo* and *in vitro* experiments.

TCM has attracted increasing attention worldwide. A broad space for the development of TCM, including its therapeutic effect on malignant tumors, is expected in the future. Chrysin, also known as aspen flavin, is a flavonoid compound extracted from *Oroxylum indicum* 29. The content of Chrysin in propolis is high, and increasing evidence reveals the role of COMP and TAGLN in the malignant progression of tumors. Therefore, COMP and TAGLN are promising new targets for the treatment of malignant tumors. Chrysin, as an interfacial inhibitor of the COMP/TAGLN complex, may regulate tumors through a variety of pathways. Chrysin exhibits antitumor effects by inhibiting cell proliferation 30, inducing apoptosis 31, eliminating reactive oxygen species, and preventing angiogenesis 32, 33, but its direct target remains unknown 34, 35. Chrysin can directly act on the COMP/TAGLN interaction interface and inhibit mesenchymal transformation, thereby hindering the malignant progression of colorectal cancer (Figure 8). The deficiency of this study is that it does not clarify whether COMP is the initiating factor of EMT, and the factors regulating COMP are unclear. These limitations need to be further strengthened in follow-up research.

## Conclusion

In this study, we found that COMP regulated tumor cytoskeletal remodeling and promoted mesenchymal transition by interacting directly with TAGLN. Chrysin inhibited EMT by inhibiting the COMP/TAGLN interaction interface and showed excellent antitumor activity. Research on the mechanism of the EMT and the structural modification of lead compounds is of great significance for the development of clinical antitumor drugs targeted at COMP/TAGLN.

## Supplementary Material

Supplementary figures and tables.

## Abbreviations

EMT: epithelial-to-mesenchymal transition
IHC: immunohistochemistry
COMP: cartilage oligomeric matrix protein
TAGLN: transgelin
DEGs: differentially expressed genes
GO: Gene Ontology
KEGG: Kyoto Encyclopedia of Genes and Genomes
MDS: Multidimensional scaling
STORM: stochastic optical reconstruction microscopy
TCM: traditional Chinese medicine
