# Supplementary material for: Cartilage Oligomeric Matrix Protein promotes epithelial-mesenchymal transition by interacting with Transgelin in Colorectal Cancer
Source: Theranostics. 2020 Jul 9;10(19):8790–806. doi: 10.7150/thno.44456 (PMC7392026; doi:10.7150/thno.44456)
Supplement: Supplementary file 1 — Supplementary figures and tables. [file thnov10p8790s1.pdf]

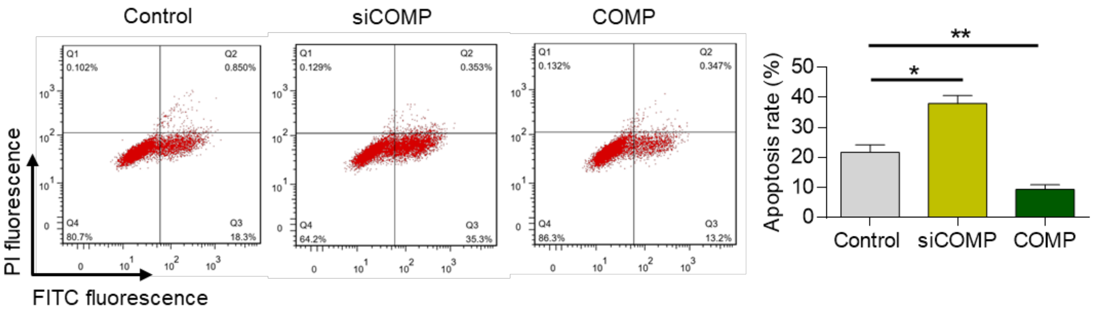

Figure S1. COMP protects from cell apoptosis. Apoptosis was induced in the SW620 cells by a 48-h incubation with brefeldin A. The experimental results showed that overexpression of COMP inhibited the apoptosis induced by Brefeldin A of SW620, while COMP knockdown promoted the apoptosis of SW620.

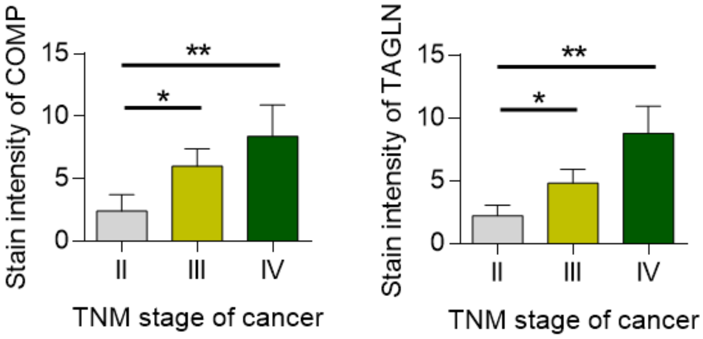

Figure S2. Patients with high expression of COMP and TAGLN had higher clinical staging.

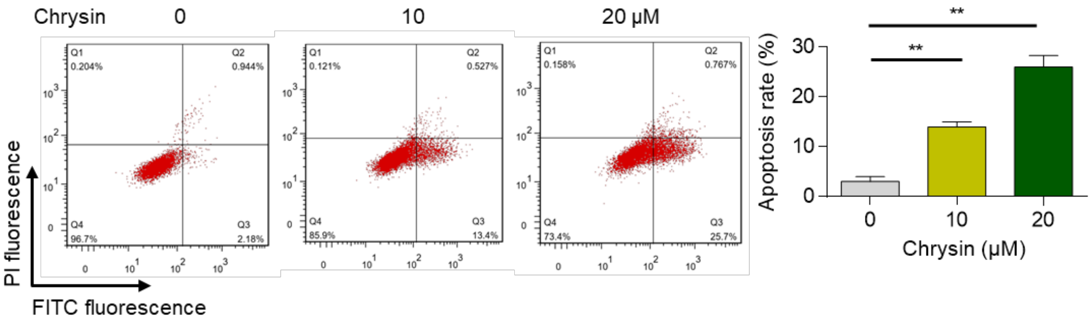

Figure S3. Chrysin can induce apoptosis of SW620 cells in a dose-dependent manner.

Table S1. Unregulated genes in colon cancer.

Genes	logFC	PValue	FDR
KLK6	2.905	1.54E-87	2.20E-84
CST1	2.287	3.47E-72	2.24E-69
FEZF1	3.752	1.18E-71	7.19E-69
SFTA2	3.107	2.13E-63	9.27E-61
KLK8	3.770	5.27E-62	2.03E-59
SPERT	3.854	5.71E-59	1.85E-56
KLK7	2.712	5.28E-56	1.43E-53
COL10A1	1.810	3.29E-55	8.34E-53
NOTUM	1.840	6.04E-55	1.48E-52
MMP7	1.575	3.39E-54	7.99E-52
CASC21	2.661	4.00E-53	8.91E-51
ZIC5	3.292	7.35E-52	1.55E-49
DKK4	2.819	9.01E-50	1.72E-47
KRT80	1.421	4.71E-49	8.51E-47
SOX14	3.151	4.68E-48	8.22E-46
CST2	2.528	5.68E-48	9.90E-46
AC123023	2.744	4.03E-47	6.73E-45
KRT75	2.993	7.75E-47	1.27E-44
SERPINA4	2.862	4.75E-46	7.56E-44
C6orf15	3.069	5.05E-46	7.97E-44
OTX1	1.903	8.69E-45	1.27E-42
IBSP	2.917	8.38E-44	1.14E-41
ESM1	1.581	1.16E-43	1.55E-41
WNT2	1.509	3.68E-42	4.64E-40
KLC3	2.477	4.59E-42	5.72E-40
FGF19	2.519	6.33E-42	7.82E-40
CST4	3.508	9.63E-42	1.16E-39
DMBX1	2.763	9.22E-41	1.05E-38
IGFL4	1.909	5.41E-40	5.92E-38
AC005256	3.497	1.24E-39	1.31E-37
C5orf46	3.080	1.94E-38	1.89E-36
BLACAT1	1.520	3.00E-38	2.88E-36
WNT7B	2.188	5.98E-38	5.73E-36
FOXQ1	1.188	6.03E-38	5.76E-36
IGFL1	3.983	9.20E-38	8.73E-36
CDH3	1.077	1.66E-37	1.57E-35
KRT6A	2.076	4.61E-37	4.25E-35
AKR1C4	2.389	1.31E-36	1.18E-34
ELF5	2.549	1.35E-36	1.20E-34
TCN1	1.521	1.92E-36	1.69E-34
CASC19	1.881	3.77E-36	3.24E-34
AC007128	1.998	7.36E-36	6.19E-34

FIRRE	1.967	1.01E-35	8.38E-34
EPYC	2.515	1.14E-35	9.33E-34
KRT6B	1.521	1.63E-35	1.32E-33
COL11A1	1.185	5.22E-35	4.15E-33
C8orf74	3.745	6.87E-35	5.38E-33
ADAM20P3	3.877	7.90E-35	6.14E-33
SP8	2.498	2.44E-33	1.77E-31
CLDN2	1.014	2.91E-33	2.10E-31
KRT23	1.145	3.21E-33	2.30E-31
GAD1	1.547	4.91E-33	3.47E-31
MMP13	1.947	5.30E-33	3.74E-31
SLCO1B3	1.750	6.08E-33	4.28E-31
COMP	1.287	2.39E-32	1.64E-30
LY6G6E	2.954	5.69E-32	3.84E-30
KRT16	1.972	1.01E-31	6.73E-30
GBX2	2.978	1.86E-31	1.23E-29
AC007099	3.504	1.95E-31	1.28E-29
MAGEA3	3.471	2.56E-31	1.68E-29
LEMD1	1.984	3.56E-31	2.31E-29
SPRR2D	3.037	3.91E-31	2.52E-29
SPRR1A	3.274	4.32E-31	2.77E-29
AP000697	3.704	7.11E-31	4.55E-29
LY6G6F	3.081	1.45E-30	9.05E-29
C6orf223	1.026	2.09E-30	1.30E-28
C2orf61	1.707	3.52E-30	2.15E-28
LY6G6D	1.487	6.99E-30	4.19E-28
SPRR1B	3.032	8.47E-30	5.03E-28
RAET1L	1.637	8.77E-30	5.18E-28
AMELX	2.596	4.26E-29	2.41E-27
CPNE7	1.031	4.96E-29	2.77E-27
DDN	1.313	6.33E-29	3.50E-27
PRSS56	2.941	1.24E-28	6.74E-27
STRA6	1.174	1.74E-28	9.30E-27
C14orf105	2.878	2.20E-28	1.18E-26
UCN2	1.887	4.60E-28	2.43E-26
PIWIL1	1.369	1.56E-27	8.01E-26
CCAT1	1.090	1.57E-27	8.05E-26
SALL4	1.269	2.21E-27	1.12E-25
PAEP	2.395	4.96E-27	2.43E-25
PDX1	1.284	6.64E-27	3.23E-25
PAH	1.528	7.97E-27	3.85E-25
MCIDAS	1.484	8.79E-27	4.22E-25
FABP6	1.124	9.63E-27	4.59E-25
AC079466	2.842	1.94E-26	9.07E-25

AC142293	2.736	3.19E-26	1.46E-24
EN2	1.793	3.71E-26	1.69E-24
FGB	2.103	4.94E-26	2.23E-24
MSLNL	2.773	5.51E-26	2.47E-24
AC007277	1.844	6.20E-26	2.76E-24
SPRR2A	2.606	6.64E-26	2.94E-24
CRNDE	1.187	8.01E-26	3.53E-24
TCF24	2.320	8.61E-26	3.78E-24
MAGEA6	3.056	1.63E-25	7.00E-24
IRX5	1.516	3.19E-25	1.35E-23
HABP2	1.885	3.21E-25	1.36E-23
OBP2B	2.942	4.19E-25	1.74E-23
TNNI3	1.488	7.57E-25	3.10E-23
HTR1D	1.061	8.60E-25	3.51E-23
KRT17	1.009	1.10E-24	4.45E-23
EPHX4	1.052	1.34E-24	5.43E-23
ZNF280A	2.851	1.38E-24	5.56E-23
PRR9	3.240	1.71E-24	6.78E-23
AC068121	2.831	4.24E-24	1.67E-22
CNTD2	1.109	4.83E-24	1.89E-22
FAM195CP	2.047	7.57E-24	2.92E-22
NPSR1	1.612	1.99E-23	7.50E-22
VGLL1	2.971	2.54E-23	9.56E-22
AC018359	2.736	4.40E-23	1.64E-21
GOLGA6L2	3.311	4.63E-23	1.72E-21
PCAT2	3.055	1.82E-22	6.64E-21
ULBP1	1.415	2.13E-22	7.73E-21
ZIC2	1.231	3.42E-22	1.22E-20
C1orf105	1.826	3.60E-22	1.28E-20
CXCL17	1.688	4.14E-22	1.46E-20
DUSP27	1.068	5.12E-22	1.80E-20
C3P1	2.718	5.73E-22	2.01E-20
NCAPD2P1	1.832	6.07E-22	2.12E-20
TPSP2	2.655	7.56E-22	2.63E-20
IL11	1.065	7.44E-21	2.43E-19
FGF20	2.007	1.14E-20	3.69E-19
NEUROG2	2.295	1.27E-20	4.08E-19
LPO	1.618	1.28E-20	4.11E-19
MMP8	1.862	1.30E-20	4.17E-19
GJB4	1.048	1.51E-20	4.82E-19
ERVMER61	2.946	2.13E-20	6.68E-19
SLC26A9	1.762	2.13E-20	6.70E-19
ONECUT3	1.605	2.18E-20	6.84E-19
SMKR1	1.150	3.27E-20	1.01E-18

SPRR3	2.278	5.99E-20	1.82E-18
TLX1	1.022	6.31E-20	1.91E-18
NXPH1	2.751	8.81E-20	2.65E-18
ULBP2	1.134	9.72E-20	2.91E-18
DUSP5P1	2.332	1.47E-19	4.32E-18
SLCO1B1	2.758	1.90E-19	5.54E-18
LY6D	1.664	2.15E-19	6.24E-18
ARHGAP40	2.087	3.08E-19	8.83E-18
MYBPHL	2.057	3.09E-19	8.86E-18
FGA	1.544	3.37E-19	9.64E-18
DSG3	1.013	4.03E-19	1.14E-17
SIX1	1.348	4.84E-19	1.37E-17
MIR31HG	2.402	5.02E-19	1.42E-17
CNN2P4	2.385	5.23E-19	1.47E-17
ASCL5	1.156	5.45E-19	1.53E-17
BTBD16	1.160	5.67E-19	1.59E-17
AC012501	2.476	1.00E-18	2.75E-17
SPATA12	1.121	1.23E-18	3.37E-17
AL162759	2.682	1.41E-18	3.84E-17
PLA2G3	1.265	2.55E-18	6.79E-17
MAGEA12	2.696	2.60E-18	6.92E-17
TRIM71	1.878	2.80E-18	7.44E-17
AC093732	1.796	3.71E-18	9.76E-17
AC093850	2.162	3.82E-18	1.01E-16
SNORA74A	2.875	4.07E-18	1.06E-16
AC012512	2.077	4.24E-18	1.11E-16
SERPINB7	1.682	5.88E-18	1.53E-16
PLAC4	1.372	6.92E-18	1.78E-16
AC004593	1.570	7.08E-18	1.82E-16
APELA	1.469	9.16E-18	2.33E-16
NXPH4	1.015	1.01E-17	2.56E-16
KRT83	1.838	1.02E-17	2.58E-16
DMRTA2	1.337	1.23E-17	3.09E-16
AKAP4	2.473	1.30E-17	3.24E-16
AC140076	2.023	1.51E-17	3.75E-16
CPA4	1.543	1.70E-17	4.22E-16
DUSP9	1.381	1.96E-17	4.82E-16
DCSTAMP	1.517	2.00E-17	4.93E-16
SLAMF9	1.368	2.94E-17	7.17E-16
AC133785	2.235	3.67E-17	8.83E-16
AC019100	1.962	3.76E-17	9.02E-16
SBSN	1.886	3.77E-17	9.03E-16
PPBP	1.123	4.96E-17	1.18E-15
CYP4F23P	1.476	5.40E-17	1.28E-15

LUCAT1	1.342	8.28E-17	1.95E-15
DMRT3	1.890	9.20E-17	2.16E-15
MIR3189	1.334	9.94E-17	2.33E-15
FAM150A	1.024	1.47E-16	3.41E-15
SNORA71A	1.303	1.49E-16	3.45E-15
HOXC11	1.442	1.55E-16	3.57E-15
S100A11P1	2.353	2.43E-16	5.55E-15
PTF1A	2.578	2.99E-16	6.79E-15
MMP20	1.915	3.21E-16	7.25E-15
BAAT	1.451	3.47E-16	7.82E-15
CCAT2	1.973	7.52E-16	1.65E-14
APOA2	1.731	8.72E-16	1.91E-14
SLC22A11	1.027	1.10E-15	2.40E-14
CHST4	1.108	1.17E-15	2.53E-14
AC004988	1.653	1.41E-15	3.04E-14
SPATA20P1	2.221	2.23E-15	4.75E-14
KDELC1P1	1.545	2.65E-15	5.62E-14
PGC	1.109	3.00E-15	6.31E-14
CLDN16	1.053	4.07E-15	8.45E-14
XIRP1	1.191	4.13E-15	8.57E-14
WFDC10A	2.036	5.41E-15	1.11E-13
IFNWP19	2.151	5.51E-15	1.12E-13
DUXAP10	1.619	5.91E-15	1.20E-13
KRT6C	2.151	8.02E-15	1.61E-13
TMEM40	1.615	8.78E-15	1.76E-13
KRT87P	1.811	1.00E-14	1.99E-13
AHSG	1.581	1.15E-14	2.29E-13
PRSS41	1.553	1.25E-14	2.47E-13
RHBG	1.778	1.67E-14	3.24E-13
RNA5SP18	2.390	1.67E-14	3.24E-13
CYCSP6	1.275	2.48E-14	4.74E-13
PRDM12	1.223	2.74E-14	5.22E-13
IFITM5	2.192	2.85E-14	5.43E-13
BBOX1	1.123	3.05E-14	5.79E-13
PGLYRP3	2.181	3.53E-14	6.64E-13
CSF2	1.151	3.98E-14	7.43E-13
RAET1K	1.331	4.35E-14	8.08E-13
BHLHA9	2.133	4.38E-14	8.13E-13
SNORD12B	1.343	4.50E-14	8.35E-13
SH2D5	1.230	5.46E-14	1.01E-12
IFNE	2.188	6.26E-14	1.15E-12
CSTL1	1.586	7.13E-14	1.30E-12
SNORA71D	2.183	7.23E-14	1.32E-12
AC064834	1.800	7.52E-14	1.37E-12

RPS18P1	1.945	7.57E-14	1.38E-12
MAGEB17	1.137	8.14E-14	1.47E-12
SNORD12C	2.028	8.35E-14	1.51E-12
ATP6V0A4	1.695	9.72E-14	1.75E-12
AC005264	1.787	1.04E-13	1.86E-12
PTTG3P	1.953	1.14E-13	2.03E-12
FOXI3	2.292	1.17E-13	2.09E-12
PCMTD1P3	1.928	1.27E-13	2.24E-12
IGFL1P1	1.994	1.97E-13	3.43E-12
CALCA	1.184	2.32E-13	4.02E-12
OFCC1	1.887	2.79E-13	4.80E-12
AC105399	2.090	2.83E-13	4.86E-12
AL589743	1.422	3.26E-13	5.58E-12
VTCN1	1.414	3.26E-13	5.58E-12
AC002331	1.561	3.58E-13	6.13E-12
TDGF1P3	1.651	3.64E-13	6.21E-12
SNORD48	1.886	4.01E-13	6.82E-12
GSDMC	1.132	4.29E-13	7.29E-12
RPL37AP8	1.163	4.62E-13	7.84E-12
TMEM75	1.548	6.48E-13	1.09E-11
IL36RN	1.804	8.41E-13	1.40E-11
SPDYC	1.050	9.32E-13	1.55E-11
BEST3	1.318	1.21E-12	1.99E-11
AFP	1.385	1.50E-12	2.46E-11
SNORA71C	1.088	1.63E-12	2.66E-11
SEC14L4	1.291	1.70E-12	2.76E-11
F2	1.157	1.79E-12	2.91E-11
OACYLP	1.136	1.84E-12	2.98E-11
HSPE1P5	1.906	2.40E-12	3.86E-11
PPP1R14BP2	1.242	2.41E-12	3.86E-11
RPL18P13	1.143	3.99E-12	6.25E-11
ALOXE3	1.243	4.02E-12	6.29E-11
GAST	2.035	4.13E-12	6.46E-11
DIRC1	1.984	4.82E-12	7.49E-11
CAMKV	1.030	7.10E-12	1.09E-10
MAGEA11	1.378	7.78E-12	1.18E-10
TECRP2	1.617	8.32E-12	1.26E-10
RPL35AP21	1.519	9.20E-12	1.39E-10
TAS2R38	1.127	9.30E-12	1.40E-10
PRDM13	1.292	1.35E-11	2.00E-10
RFX8	1.128	2.39E-11	3.48E-10
SNRPFP1	1.518	2.55E-11	3.70E-10
MAGOH3P	1.538	2.63E-11	3.80E-10
NAT16	1.324	3.57E-11	5.09E-10

TPT1P12	1.262	3.91E-11	5.55E-10
FGG	1.787	4.18E-11	5.91E-10
RPS3AP46	1.465	4.34E-11	6.13E-10
ORM1	1.269	4.38E-11	6.18E-10
S100A7	1.698	4.51E-11	6.35E-10
CSAG1	1.711	4.84E-11	6.79E-10
AC009362	1.070	5.03E-11	7.04E-10
MIR7111	1.214	5.74E-11	7.98E-10
HULC	1.595	6.41E-11	8.87E-10
SMCO2	1.081	6.42E-11	8.87E-10
AC018641	1.325	7.08E-11	9.73E-10
METTL11B	1.879	7.20E-11	9.88E-10
SAA4	1.666	7.76E-11	1.06E-09
HBE1	1.652	7.97E-11	1.09E-09
CST5	1.906	9.07E-11	1.23E-09
AC009410	1.212	9.91E-11	1.34E-09
AC104297	1.093	1.14E-10	1.53E-09
MIOX	1.116	1.15E-10	1.54E-09
IL17C	1.084	1.21E-10	1.62E-09
AC112721	1.666	1.50E-10	1.99E-09
SCDP1	1.332	1.52E-10	2.02E-09
GRK1	1.073	1.66E-10	2.19E-09
SNORA23	1.517	1.66E-10	2.19E-09
LRRN4	1.017	2.21E-10	2.89E-09
ATP5G1P6	1.667	2.71E-10	3.51E-09
SNORD88A	1.611	3.15E-10	4.05E-09
HIST1H2AH	1.286	3.23E-10	4.14E-09
UBE2SP2	1.032	3.28E-10	4.19E-09
SLCO1A2	1.225	3.39E-10	4.32E-09
C9orf57	1.865	3.52E-10	4.48E-09
SNORA80B	1.370	3.63E-10	4.61E-09
SNORD72	1.648	3.63E-10	4.61E-09
RPL36P16	1.415	3.98E-10	5.03E-09
RYKP1	1.129	4.30E-10	5.42E-09
AQP2	1.740	4.58E-10	5.76E-09
RPS24P16	1.436	4.77E-10	5.98E-09
SCARNA5	1.265	5.63E-10	7.03E-09
TMEM249	1.019	5.96E-10	7.42E-09
AC118754	1.378	6.44E-10	7.98E-09
FAR2P1	1.176	6.59E-10	8.14E-09
PCAT14	1.042	7.25E-10	8.93E-09
CLLU1	1.576	7.56E-10	9.29E-09
HIST1H4B	1.317	8.18E-10	1.00E-08
HCRT	1.408	8.37E-10	1.02E-08

GTF2F2P1	1.359	8.50E-10	1.04E-08
AKR1C8P	1.598	1.03E-09	1.25E-08
UPK2	1.161	1.19E-09	1.43E-08
POU3F2	1.286	1.36E-09	1.63E-08
WASIR2	1.196	1.55E-09	1.85E-08
GNGT1	1.067	1.58E-09	1.88E-08
AC144450	1.183	1.70E-09	2.01E-08
HIST1H3J	1.115	2.42E-09	2.82E-08
AC092573	1.012	2.45E-09	2.85E-08
DDX3P1	1.166	2.73E-09	3.17E-08
RPL7AP4	1.135	2.79E-09	3.23E-08
PRDX2P4	1.295	2.94E-09	3.40E-08
IZUMO2	1.065	3.39E-09	3.89E-08
HIST1H2AB	1.418	3.41E-09	3.91E-08
PDCD5P1	1.254	3.57E-09	4.09E-08
EIF3IP1	1.399	4.29E-09	4.87E-08
MRPL40P1	1.652	4.41E-09	5.01E-08
HIST1H2BI	1.453	4.73E-09	5.34E-08
AC013439	1.270	4.77E-09	5.38E-08
TCAM1P	1.330	5.05E-09	5.68E-08
SULT1C2P1	1.246	5.14E-09	5.77E-08
LRP2	1.281	5.58E-09	6.21E-08
HES7	1.213	6.94E-09	7.63E-08
PPEF1	1.208	1.00E-08	1.08E-07
ERHP1	1.178	1.30E-08	1.38E-07
HIST1H3I	1.334	1.67E-08	1.75E-07
AC105009	1.517	1.72E-08	1.80E-07
RN7SL28P	1.475	1.73E-08	1.81E-07
HSPD1P2	1.395	2.40E-08	2.49E-07
MIR6797	1.282	3.02E-08	3.09E-07
TEX19	1.130	3.42E-08	3.48E-07
FGF8	1.300	3.49E-08	3.54E-07
AC005592	1.153	3.92E-08	3.96E-07
C15orf54	1.407	4.02E-08	4.04E-07
UROC1	1.399	4.02E-08	4.04E-07
AL022326	1.154	4.16E-08	4.18E-07
RPL31P2	1.203	4.25E-08	4.26E-07
AC079145	1.043	4.41E-08	4.41E-07
POLD2P1	1.263	4.78E-08	4.76E-07
IFNL3P1	1.090	5.09E-08	5.03E-07
PCBP2P1	1.262	5.86E-08	5.76E-07
PKMP5	1.045	5.92E-08	5.81E-07
BHMG1	1.433	6.03E-08	5.91E-07
RPS8P3	1.249	6.49E-08	6.32E-07

KLK5	1.440	7.65E-08	7.38E-07
ANKRD33	1.253	8.66E-08	8.29E-07
SEPHS1P4	1.160	9.99E-08	9.49E-07
EPHA8	1.343	1.06E-07	1.00E-06
EEF1DP5	1.193	1.08E-07	1.02E-06
IGFL3	1.078	1.09E-07	1.03E-06
ANKRD1	1.091	1.31E-07	1.22E-06
ARF1P2	1.296	1.48E-07	1.37E-06
SNRPD2P1	1.238	1.86E-07	1.71E-06
FDPSP8	1.166	1.88E-07	1.72E-06
AC064850	1.009	2.15E-07	1.96E-06
SNRPCP2	1.033	2.21E-07	2.01E-06
AP000577	1.258	2.22E-07	2.01E-06
ANKRD20A19P	1.360	2.31E-07	2.09E-06
SUPT4H1P	1.412	2.63E-07	2.36E-06
AC092415	1.410	2.86E-07	2.56E-06
SLC25A24P1	1.157	2.90E-07	2.59E-06
OBP2A	1.301	3.11E-07	2.77E-06
RPL37P1	1.267	3.56E-07	3.14E-06
SERPINH1P1	1.163	4.45E-07	3.88E-06
MUCL1	1.006	4.72E-07	4.10E-06
TREML3P	1.194	5.35E-07	4.62E-06
AC012066	1.003	5.87E-07	5.04E-06
ILF2P1	1.167	6.32E-07	5.41E-06
AC019129	1.001	6.73E-07	5.74E-06
MPP4	1.051	7.88E-07	6.66E-06
AC007790	1.209	8.94E-07	7.50E-06
RNU4ATAC	1.056	9.05E-07	7.58E-06
EN1	1.215	9.58E-07	8.00E-06
FDPSP5	1.240	9.72E-07	8.11E-06
KAAG1	1.179	1.06E-06	8.83E-06
FKBP4P6	1.268	1.11E-06	9.15E-06
PRDX2P1	1.146	1.13E-06	9.30E-06
EIF5AP3	1.184	1.22E-06	1.01E-05
AC005102	1.098	1.57E-06	1.27E-05
HIST2H2AB	1.145	1.61E-06	1.30E-05
RPS7P15	1.075	1.76E-06	1.41E-05
NUFIP1P	1.216	1.97E-06	1.57E-05
AC007750	1.195	1.97E-06	1.57E-05
GCM1	1.023	2.01E-06	1.59E-05
EPGN	1.288	2.03E-06	1.61E-05
CHRNA6	1.024	2.10E-06	1.66E-05
HBQ1	1.014	2.20E-06	1.74E-05
ACTBL2	1.209	2.44E-06	1.91E-05

PMCH	1.191	2.71E-06	2.10E-05
MIR6835	1.041	2.82E-06	2.18E-05
SLED1	1.081	2.84E-06	2.19E-05
HNRNPH1P3	1.180	3.55E-06	2.70E-05
KLF2P1	1.186	4.39E-06	3.30E-05
OR7E85P	1.176	4.40E-06	3.31E-05
AC073326	1.208	5.15E-06	3.82E-05
AC024995	1.100	5.32E-06	3.93E-05
IFIT1P1	1.166	5.60E-06	4.12E-05
ZDHHC4P1	1.114	6.46E-06	4.70E-05
PARP1P1	1.045	6.62E-06	4.82E-05
TMEM213	1.012	6.83E-06	4.96E-05
GPRC6A	1.004	7.03E-06	5.09E-05
GAPDHP67	1.019	7.09E-06	5.13E-05
SCARNA8	1.111	7.29E-06	5.27E-05
RPL7P33	1.102	8.08E-06	5.79E-05
SIX3	1.185	8.28E-06	5.92E-05
SNORD1B	1.089	1.55E-05	0.000107
EGOT	1.099	1.74E-05	0.000119
SEPW1P	1.063	1.81E-05	0.000124
CD300LD	1.143	1.86E-05	0.000127
AC012354	1.021	3.15E-05	0.000207
RNY1	1.030	3.48E-05	0.000226
AC023672	1.042	3.82E-05	0.000247
NPM1P40	1.010	5.37E-05	0.000337
IRG1	1.006	0.000113	0.000664

Table S2. Downregulated genes in colon cancer.

Genes	logFC	PValue	FDR
OTOP2	-2.658	2.42E-179	4.84E-175
CLDN8	-2.102	1.11E-110	1.11E-106
MYOC	-2.397	2.53E-109	1.69E-105
CD177P1	-2.668	4.33E-109	2.17E-105
TMIGD1	-1.849	5.71E-107	2.29E-103
LGI1	-2.586	7.85E-103	2.62E-99
GUCA2B	-1.752	5.08E-96	1.45E-92
SLC17A8	-2.557	1.87E-95	4.67E-92
PCSK2	-2.198	7.00E-94	1.56E-90
DAO	-2.296	2.98E-90	5.96E-87
PYY	-1.719	2.76E-89	5.02E-86
MS4A10	-2.597	7.42E-88	1.24E-84
GCG	-1.800	1.17E-87	1.80E-84
RERGL	-2.144	5.87E-87	7.84E-84
CA1	-1.459	7.26E-87	9.09E-84

SLC30A10	-1.886	2.17E-86	2.56E-83
NPY2R	-2.466	8.85E-85	9.85E-82
NGB	-2.563	3.14E-84	3.32E-81
PHOX2B	-2.284	1.80E-83	1.80E-80
INSL5	-2.149	3.75E-83	3.58E-80
RN7SKP127	-2.595	8.55E-83	7.78E-80
HSD3B2	-1.951	2.34E-81	2.04E-78
SST	-1.860	3.07E-80	2.56E-77
AQP8	-1.364	1.86E-76	1.49E-73
VSTM2A	-2.338	3.74E-76	2.88E-73
PMP2	-2.251	2.04E-74	1.51E-71
CMTM5	-2.347	1.94E-73	1.39E-70
KHDRBS2	-2.403	9.27E-73	6.40E-70
FEV	-2.039	2.29E-72	1.53E-69
FAM180B	-2.385	3.82E-72	2.39E-69
BRINP3	-1.714	4.01E-71	2.36E-68
NRXN1	-1.763	1.26E-70	7.23E-68
FAM135B	-1.930	7.83E-70	4.36E-67
STMN4	-2.170	1.34E-69	7.24E-67
C14orf180	-2.377	1.52E-68	8.00E-66
SGCG	-2.228	5.32E-68	2.73E-65
ASB5	-2.155	1.47E-66	7.37E-64
TMEFF2	-2.131	1.94E-66	9.50E-64
ANGPTL7	-1.886	2.79E-66	1.33E-63
PLP1	-1.536	4.65E-66	2.17E-63
SCGN	-1.528	6.41E-65	2.92E-62
CMA1	-1.840	1.58E-63	7.02E-61
SLC5A7	-2.080	2.22E-63	9.46E-61
SLC7A14	-1.942	2.79E-63	1.16E-60
SPOCK3	-2.109	1.38E-62	5.64E-60
SLC6A19	-1.471	2.42E-62	9.58E-60
AC007182	-1.737	2.44E-62	9.58E-60
SLITRK3	-1.878	8.03E-62	3.04E-59
NEUROD1	-1.905	1.33E-61	4.92E-59
ABCB11	-1.738	1.37E-60	4.98E-58
CA7	-1.276	1.53E-60	5.48E-58
MS4A12	-1.248	1.74E-60	6.13E-58
SYT10	-2.233	8.37E-60	2.89E-57
AC106869	-2.021	1.07E-59	3.63E-57
SEMA3E	-1.651	1.14E-59	3.81E-57
BMP3	-1.338	2.25E-59	7.38E-57
GALR1	-2.207	1.10E-58	3.50E-56
XKR4	-1.757	2.65E-58	8.29E-56
TCEAL5	-2.136	3.40E-58	1.05E-55

GABRG2	-2.150	6.72E-58	2.04E-55
CHST9	-2.157	8.18E-58	2.44E-55
SCN7A	-1.425	2.55E-57	7.52E-55
PI16	-1.411	2.98E-57	8.65E-55
CLCA4	-1.129	7.32E-57	2.09E-54
P2RX2	-1.960	2.14E-56	6.04E-54
ASTN1	-1.843	2.40E-56	6.67E-54
RIMS4	-1.779	3.37E-56	9.26E-54
TUSC5	-1.942	1.01E-55	2.71E-53
CDH19	-1.552	1.16E-55	3.06E-53
MGAT4C	-2.049	1.60E-55	4.17E-53
ST8SIA3	-2.123	2.65E-55	6.81E-53
PRIMA1	-1.411	3.53E-55	8.84E-53
HS3ST6	-2.170	4.09E-55	1.01E-52
PHOX2A	-2.003	9.40E-55	2.27E-52
HTR3C	-2.078	1.07E-54	2.56E-52
C2orf40	-1.530	4.40E-54	1.02E-51
NPY6R	-1.507	4.94E-54	1.14E-51
NPY	-1.917	1.74E-53	3.97E-51
SYT4	-1.760	2.53E-53	5.68E-51
PIRT	-1.533	7.48E-53	1.65E-50
RXRG	-1.672	1.55E-52	3.37E-50
CD177	-1.075	3.48E-52	7.50E-50
CA4	-1.118	5.79E-52	1.23E-49
HMP19	-1.512	7.69E-52	1.61E-49
MORN5	-1.890	8.01E-52	1.66E-49
MUSK	-1.661	1.50E-51	3.07E-49
BEST4	-1.182	1.63E-51	3.29E-49
CHGA	-1.119	1.36E-50	2.72E-48
ADCYAP1R1	-1.551	1.38E-50	2.73E-48
FIGF	-1.537	1.88E-50	3.70E-48
PCAT18	-2.089	2.31E-50	4.50E-48
GPM6A	-1.419	3.34E-50	6.44E-48
OLFM3	-1.880	9.25E-50	1.75E-47
MYOT	-1.585	1.29E-49	2.42E-47
ELANE	-1.837	1.38E-49	2.56E-47
TCEAL2	-1.547	2.11E-49	3.87E-47
CNTN2	-1.541	4.47E-49	8.15E-47
LMX1A	-1.870	6.18E-49	1.10E-46
DPP6	-1.451	1.27E-48	2.25E-46
SFRP5	-1.557	7.07E-48	1.22E-45
ZG16	-1.012	9.04E-48	1.55E-45
AC074363	-2.029	2.69E-47	4.57E-45
GBA3	-1.271	3.38E-47	5.69E-45

RSPO2	-1.365	5.31E-47	8.79E-45
ADH1B	-1.108	2.27E-46	3.70E-44
CHRM2	-1.418	3.30E-46	5.32E-44
CASR	-1.963	3.49E-46	5.60E-44
AMPD1	-1.399	7.39E-46	1.16E-43
ATP1A2	-1.193	1.06E-45	1.65E-43
B4GALNT2	-1.092	1.21E-45	1.87E-43
CTNND2	-1.517	2.56E-45	3.92E-43
SERPINA9	-1.928	3.88E-45	5.88E-43
CD300LG	-1.744	5.00E-45	7.54E-43
SLC18A3	-1.990	6.13E-45	9.16E-43
CLVS2	-1.883	8.04E-45	1.19E-42
CWH43	-1.173	8.23E-45	1.21E-42
ABCB5	-1.720	8.85E-45	1.28E-42
UGT1A8	-1.231	1.05E-44	1.51E-42
ELAVL3	-1.810	1.24E-44	1.77E-42
ABCA8	-1.132	1.35E-44	1.92E-42
NRSN1	-1.884	2.64E-44	3.73E-42
KIAA2022	-1.477	3.48E-44	4.85E-42
AL928768	-1.519	3.49E-44	4.85E-42
DMRTA1	-1.439	4.07E-44	5.62E-42
CNGB1	-1.692	4.50E-44	6.18E-42
WISP2	-1.244	8.74E-44	1.18E-41
SCNN1G	-1.417	1.74E-43	2.33E-41
SFRP1	-1.104	1.83E-43	2.43E-41
AC004947	-1.760	5.45E-43	7.18E-41
SNAP91	-1.566	6.23E-43	8.15E-41
KRT9	-1.940	8.30E-43	1.08E-40
MAB21L1	-1.605	1.04E-42	1.35E-40
BCHE	-1.229	1.30E-42	1.67E-40
GSG1L	-1.870	2.22E-42	2.83E-40
ATCAY	-1.571	3.56E-42	4.51E-40
CASQ2	-1.225	3.84E-42	4.80E-40
PLCXD3	-1.513	6.88E-42	8.43E-40
SLC27A6	-1.627	6.90E-42	8.43E-40
OGN	-1.122	8.12E-42	9.86E-40
ANGPTL5	-1.969	1.02E-41	1.23E-39
NUS1P2	-1.964	1.17E-41	1.39E-39
TAGLN3	-1.507	1.53E-41	1.81E-39
BAI3	-1.430	1.60E-41	1.88E-39
ATP2B3	-1.870	2.87E-41	3.37E-39
UG0898H09	-1.571	3.78E-41	4.40E-39
FRRS1L	-1.486	6.83E-41	7.90E-39
LHFPL4	-1.510	7.46E-41	8.59E-39

CADM3	-1.299	8.34E-41	9.55E-39
NEFM	-1.498	1.12E-40	1.26E-38
GPR15	-1.227	1.97E-40	2.22E-38
BEST2	-1.124	2.51E-40	2.80E-38
NEFL	-1.295	3.41E-40	3.80E-38
PKHD1L1	-1.320	3.50E-40	3.87E-38
CNR1	-1.244	5.33E-40	5.87E-38
SLC30A8	-1.888	5.65E-40	6.15E-38
SORCS1	-1.239	1.05E-39	1.14E-37
EPHA6	-1.702	1.11E-39	1.19E-37
SPIB	-1.030	1.23E-39	1.31E-37
SLC4A10	-1.270	1.82E-39	1.92E-37
CNGA3	-1.380	2.33E-39	2.43E-37
CTNNA3	-1.390	3.10E-39	3.21E-37
RASGEF1C	-1.583	3.55E-39	3.67E-37
MEP1B	-1.165	4.36E-39	4.48E-37
FOXD3	-1.657	5.20E-39	5.32E-37
SCN2B	-1.337	6.12E-39	6.22E-37
PRPH	-1.209	7.88E-39	7.98E-37
NAP1L2	-1.264	9.43E-39	9.49E-37
COL19A1	-1.659	1.24E-38	1.24E-36
FMN2	-1.319	1.35E-38	1.35E-36
MAPK4	-1.341	1.42E-38	1.41E-36
P2RY4	-1.586	1.50E-38	1.48E-36
SLAMF6P1	-1.409	1.62E-38	1.60E-36
CTSG	-1.207	1.70E-38	1.67E-36
SCN11A	-1.373	2.35E-38	2.27E-36
SPINK2	-1.389	2.03E-37	1.91E-35
PCDH10	-1.495	3.03E-37	2.84E-35
KCTD4	-1.534	3.33E-37	3.11E-35
HSPB3	-1.435	4.27E-37	3.96E-35
HRK	-1.520	5.66E-37	5.20E-35
NR1H4	-1.164	7.46E-37	6.82E-35
LCN6	-1.660	8.99E-37	8.18E-35
LONRF2	-1.243	9.29E-37	8.42E-35
ANGPTL1	-1.095	1.15E-36	1.04E-34
DPYSL5	-1.547	1.35E-36	1.20E-34
AGTR1	-1.254	1.44E-36	1.28E-34
SLCO4C1	-1.231	2.01E-36	1.77E-34
KCNB1	-1.270	2.17E-36	1.90E-34
NPTX1	-1.160	2.26E-36	1.97E-34
HAND1	-1.325	2.31E-36	2.00E-34
SCG3	-1.266	3.50E-36	3.02E-34
PTPRZ1	-1.294	3.81E-36	3.26E-34

SLC6A15	-1.576	5.00E-36	4.26E-34
CA10	-1.585	5.22E-36	4.43E-34
TMEM179	-1.389	6.35E-36	5.37E-34
IGLJ1	-1.825	7.70E-36	6.45E-34
ADH1A	-1.844	8.50E-36	7.10E-34
C20orf166	-1.335	9.61E-36	7.98E-34
HMX2	-1.764	1.08E-35	8.89E-34
KCNA1	-1.737	1.32E-35	1.08E-33
ASPA	-1.146	1.36E-35	1.11E-33
IGKV1OR10	-1.801	1.65E-35	1.34E-33
SH2D7	-1.179	2.03E-35	1.64E-33
IGHV3OR16	-1.556	3.26E-35	2.61E-33
ZMAT4	-1.797	3.77E-35	3.01E-33
GRIN2A	-1.419	5.37E-35	4.25E-33
TEX11	-1.174	5.70E-35	4.49E-33
C11orf86	-1.035	5.81E-35	4.56E-33
MEP1AP4	-1.542	7.76E-35	6.05E-33
C16orf89	-1.103	8.34E-35	6.45E-33
TMEM100	-1.081	9.41E-35	7.25E-33
TNFRSF13B	-1.207	1.24E-34	9.53E-33
ENPP6	-1.236	1.89E-34	1.44E-32
SYT9	-1.804	2.13E-34	1.62E-32
SVOPL	-1.342	2.47E-34	1.87E-32
FCER2	-1.250	2.82E-34	2.13E-32
ADRA1A	-1.572	4.60E-34	3.47E-32
UNC5D	-1.455	6.29E-34	4.70E-32
SLITRK2	-1.535	1.02E-33	7.61E-32
TMEM151B	-1.343	1.16E-33	8.61E-32
BEX1	-1.312	1.18E-33	8.73E-32
ENAM	-1.364	1.36E-33	9.99E-32
HMGCLL1	-1.429	1.61E-33	1.18E-31
PCOLCE2	-1.092	2.00E-33	1.46E-31
PROKR1	-1.754	2.15E-33	1.57E-31
CNTFR	-1.381	3.07E-33	2.21E-31
IGKV1OR22	-1.451	3.36E-33	2.40E-31
KIF1A	-1.096	3.49E-33	2.48E-31
MOBP	-1.410	8.71E-33	6.10E-31
TUBB4A	-1.173	9.15E-33	6.39E-31
UGT1A1	-1.259	9.18E-33	6.39E-31
HTR4	-1.133	1.51E-32	1.05E-30
ANO5	-1.026	2.02E-32	1.39E-30
GLP2R	-1.025	2.06E-32	1.41E-30
ADRB3	-1.604	3.01E-32	2.05E-30
ALK	-1.469	3.03E-32	2.06E-30

CALY	-1.352	3.44E-32	2.33E-30
CELA3B	-1.607	7.04E-32	4.73E-30
INA	-1.193	7.83E-32	5.25E-30
AP001627	-1.413	1.56E-31	1.04E-29
FCRL4	-1.413	1.68E-31	1.11E-29
ZBTB16	-1.136	2.84E-31	1.85E-29
FXYD1	-1.176	2.87E-31	1.87E-29
PDZRN4	-1.081	3.58E-31	2.32E-29
GFRA2	-1.035	7.83E-31	4.99E-29
GAP43	-1.079	8.13E-31	5.17E-29
TPH1	-1.024	1.28E-30	8.07E-29
IGKV3OR2	-1.401	1.30E-30	8.16E-29
HTR3A	-1.270	1.43E-30	9.00E-29
LCN10	-1.631	1.56E-30	9.73E-29
HAND2	-1.002	1.87E-30	1.17E-28
CPB1	-1.551	2.74E-30	1.69E-28
CLEC4G	-1.315	3.41E-30	2.10E-28
IGHJ3P	-1.269	3.52E-30	2.15E-28
MAL	-1.078	4.11E-30	2.50E-28
POU3F3	-1.409	4.39E-30	2.66E-28
DIRAS2	-1.396	4.67E-30	2.83E-28
ELAVL4	-1.133	4.94E-30	2.98E-28
IGHV6	-1.094	5.96E-30	3.59E-28
SEZ6L	-1.271	7.71E-30	4.61E-28
SVOP	-1.298	7.97E-30	4.75E-28
SNTG2	-1.503	8.68E-30	5.15E-28
CCL23	-1.152	9.32E-30	5.49E-28
IGKV6D	-1.351	1.03E-29	6.08E-28
AQPEP	-1.245	1.07E-29	6.25E-28
ZNF536	-1.128	1.14E-29	6.65E-28
KLHL34	-1.173	1.23E-29	7.14E-28
TNNT3	-1.462	1.43E-29	8.31E-28
HPSE2	-1.039	1.75E-29	1.01E-27
DUSP26	-1.173	2.20E-29	1.26E-27
GNG13	-1.250	2.77E-29	1.59E-27
SYNDIG1L	-1.475	3.52E-29	2.00E-27
HTR3E	-1.714	3.53E-29	2.00E-27
CDH22	-1.522	4.21E-29	2.38E-27
AICDA	-1.614	4.33E-29	2.44E-27
GLRA4	-1.157	4.56E-29	2.56E-27
ZNF676	-1.570	4.59E-29	2.57E-27
CUX2	-1.454	5.15E-29	2.87E-27
FAM181B	-1.296	6.16E-29	3.42E-27
SLC17A1	-1.428	7.00E-29	3.86E-27

FAM163B	-1.248	7.03E-29	3.87E-27
OMD	-1.076	9.09E-29	4.99E-27
WSCD2	-1.173	1.23E-28	6.68E-27
CXCR5	-1.410	1.34E-28	7.27E-27
TMEM59L	-1.099	1.46E-28	7.88E-27
AMER3	-1.411	1.54E-28	8.32E-27
ANO3	-1.453	1.72E-28	9.24E-27
RPH3A	-1.385	3.26E-28	1.74E-26
RIC3	-1.140	3.81E-28	2.02E-26
SMYD1	-1.225	5.23E-28	2.75E-26
PCP4L1	-1.154	5.28E-28	2.77E-26
BEND4	-1.225	6.96E-28	3.64E-26
P2RY12	-1.147	7.34E-28	3.83E-26
UGT1A9	-1.650	7.73E-28	4.02E-26
GNG8	-1.437	9.48E-28	4.91E-26
C10orf105	-1.637	9.49E-28	4.91E-26
ACSM5	-1.191	1.19E-27	6.17E-26
GP2	-1.045	1.20E-27	6.20E-26
WDR17	-1.186	1.23E-27	6.32E-26
CBLN2	-1.101	2.16E-27	1.10E-25
DPEP3	-1.442	2.41E-27	1.22E-25
FGFBP2	-1.186	2.84E-27	1.43E-25
IGHV4OR15	-1.646	2.84E-27	1.43E-25
CHST8	-1.484	3.01E-27	1.51E-25
KCNE2	-1.294	3.27E-27	1.64E-25
KCNK3	-1.014	3.85E-27	1.91E-25
GFI1B	-1.145	4.00E-27	1.98E-25
TCL1A	-1.150	4.05E-27	1.99E-25
CRHBP	-1.257	6.31E-27	3.08E-25
CLEC9A	-1.189	6.65E-27	3.23E-25
SFTA1P	-1.289	7.63E-27	3.69E-25
NRG2	-1.190	8.25E-27	3.97E-25
SH3GL2	-1.220	8.93E-27	4.28E-25
ERVH	-1.328	9.63E-27	4.59E-25
IGKV1OR2	-1.448	1.17E-26	5.55E-25
KCNK2	-1.230	1.26E-26	5.96E-25
NTNG1	-1.275	1.37E-26	6.45E-25
DRD5	-1.414	1.78E-26	8.37E-25
COL6A5	-1.270	2.17E-26	1.01E-24
ANKS1B	-1.144	2.45E-26	1.14E-24
NOVA1	-1.090	2.51E-26	1.17E-24
METTL24	-1.106	2.90E-26	1.34E-24
CLCA2	-1.155	2.96E-26	1.36E-24
FLJ38122	-1.459	2.99E-26	1.38E-24

MAMDC2	-1.016	3.13E-26	1.44E-24
RPL15P21	-1.390	3.39E-26	1.55E-24
PPP1R1A	-1.035	3.83E-26	1.74E-24
ASCL1	-1.618	4.09E-26	1.85E-24
CACNG7	-1.627	4.68E-26	2.12E-24
SEZ6	-1.264	4.86E-26	2.19E-24
CELF4	-1.189	5.28E-26	2.37E-24
TMEM229A	-1.466	5.54E-26	2.48E-24
IGHV3OR15	-1.141	6.00E-26	2.68E-24
VWC2	-1.212	9.07E-26	3.98E-24
AKR1B10P1	-1.345	9.09E-26	3.98E-24
PENK	-1.173	1.28E-25	5.57E-24
TRDV1	-1.389	1.41E-25	6.13E-24
IGLV5	-1.137	1.45E-25	6.26E-24
ZNF492	-1.349	1.46E-25	6.28E-24
SCGB2A1	-1.045	2.11E-25	9.06E-24
VIT	-1.055	2.56E-25	1.10E-23
SLC17A7	-1.049	2.81E-25	1.20E-23
LIPC	-1.023	3.25E-25	1.37E-23
LRAT	-1.273	3.64E-25	1.52E-23
IGKV2OR22	-1.226	3.67E-25	1.54E-23
ERBB4	-1.550	3.91E-25	1.63E-23
DNER	-1.022	4.28E-25	1.78E-23
KCNS2	-1.352	4.72E-25	1.94E-23
RFX6	-1.199	4.74E-25	1.95E-23
GRIA1	-1.280	7.83E-25	3.20E-23
TMEM72	-1.065	9.55E-25	3.88E-23
CASC18	-1.373	1.02E-24	4.13E-23
OR2W3	-1.206	1.41E-24	5.64E-23
NPY5R	-1.392	1.59E-24	6.35E-23
UNC80	-1.341	1.68E-24	6.72E-23
IGKV5	-1.019	2.19E-24	8.69E-23
NAP1L6	-1.577	2.56E-24	1.01E-22
KCNG3	-1.126	2.88E-24	1.14E-22
LGI3	-1.233	4.78E-24	1.87E-22
ZIC1	-1.523	4.86E-24	1.89E-22
NOS1	-1.055	5.47E-24	2.13E-22
HNRNPA1P33	-1.560	5.48E-24	2.13E-22
CRABP1	-1.075	6.12E-24	2.37E-22
KRT1	-1.182	7.48E-24	2.89E-22
HMX3	-1.339	7.91E-24	3.05E-22
C1orf177	-1.107	8.97E-24	3.45E-22
KIAA1045	-1.197	9.21E-24	3.54E-22
FCRL1	-1.091	9.64E-24	3.69E-22

TMEM132C	-1.036	9.77E-24	3.73E-22
OR2S1P	-1.123	1.05E-23	3.99E-22
CPNE4	-1.192	1.06E-23	4.02E-22
GRIA4	-1.071	1.26E-23	4.78E-22
HS6ST3	-1.293	1.57E-23	5.95E-22
RSPO1	-1.109	1.68E-23	6.35E-22
SCRG1	-1.075	2.23E-23	8.39E-22
GPR88	-1.257	2.75E-23	1.03E-21
NLGN1	-1.492	4.35E-23	1.62E-21
CNTNAP3	-1.035	1.29E-22	4.74E-21
SSTR3	-1.147	1.33E-22	4.86E-21
ZNF727	-1.327	1.75E-22	6.37E-21
IGKV1D	-1.165	2.16E-22	7.80E-21
SIGLEC6	-1.042	2.36E-22	8.51E-21
NWD2	-1.029	2.40E-22	8.63E-21
AC104699	-1.138	2.71E-22	9.71E-21
SULT4A1	-1.052	3.13E-22	1.12E-20
PRAMENP	-1.252	3.71E-22	1.32E-20
DPP10	-1.306	3.89E-22	1.38E-20
CCDC129	-1.087	4.92E-22	1.73E-20
HTR7	-1.044	5.15E-22	1.81E-20
IGHV1OR21	-1.184	5.37E-22	1.89E-20
CYP4B1	-1.002	7.61E-22	2.64E-20
RBFOX1	-1.075	7.79E-22	2.70E-20
C8orf46	-1.199	8.71E-22	3.01E-20
FADS6	-1.040	8.91E-22	3.07E-20
GRIA2	-1.485	1.14E-21	3.93E-20
ABCC8	-1.016	1.31E-21	4.47E-20
GPR142	-1.382	1.36E-21	4.65E-20
KCNB2	-1.351	1.43E-21	4.87E-20
SLC5A11	-1.032	1.69E-21	5.74E-20
NRAP	-1.070	2.22E-21	7.51E-20
RPL10L	-1.026	2.25E-21	7.58E-20
PLA2G2C	-1.422	2.65E-21	8.92E-20
FAM218A	-1.186	2.68E-21	9.02E-20
SLC5A8	-1.300	2.94E-21	9.85E-20
MT1DP	-1.104	3.04E-21	1.01E-19
GRIA3	-1.019	4.22E-21	1.40E-19
CRYBA2	-1.017	4.77E-21	1.58E-19
FAR2P2	-1.117	5.77E-21	1.90E-19
NLRP7	-1.087	8.40E-21	2.74E-19
TMEM132D	-1.125	1.07E-20	3.48E-19
TAS1R1	-1.236	1.15E-20	3.71E-19
NPAS4	-1.242	1.30E-20	4.16E-19

FNDC9	-1.238	1.30E-20	4.16E-19
PTCHD1	-1.205	1.74E-20	5.53E-19
SNCB	-1.418	1.95E-20	6.17E-19
MT1P3	-1.480	2.10E-20	6.62E-19
ACADL	-1.398	2.12E-20	6.67E-19
DKFZp779M0652	-1.328	2.21E-20	6.91E-19
SPX	-1.325	2.99E-20	9.28E-19
AF131217	-1.061	3.14E-20	9.71E-19
KCNIP1	-1.218	3.71E-20	1.14E-18
KCNJ16	-1.147	4.30E-20	1.32E-18
CSMD1	-1.172	4.45E-20	1.36E-18
CLCNKB	-1.218	4.59E-20	1.40E-18
CLEC17A	-1.060	5.22E-20	1.59E-18
DACH2	-1.373	5.76E-20	1.75E-18
TAT	-1.174	6.08E-20	1.84E-18
KLRF1	-1.079	6.39E-20	1.93E-18
IL1RAPL1	-1.420	7.79E-20	2.35E-18
STXBP5L	-1.077	8.08E-20	2.43E-18
CYP11A1	-1.056	1.30E-19	3.86E-18
MIR8071	-1.373	1.35E-19	3.97E-18
SLC8A3	-1.047	1.44E-19	4.25E-18
TRIM55	-1.426	1.51E-19	4.43E-18
SCTR	-1.027	1.78E-19	5.18E-18
LIX1	-1.266	2.48E-19	7.16E-18
GALNT13	-1.046	2.63E-19	7.56E-18
AARD	-1.056	2.89E-19	8.32E-18
CCDC178	-1.159	6.58E-19	1.83E-17
AC096579	-1.191	7.09E-19	1.97E-17
LEP	-1.162	7.18E-19	1.99E-17
NOL4	-1.051	1.22E-18	3.35E-17
CYP4F29P	-1.050	1.30E-18	3.56E-17
MYO3A	-1.062	1.83E-18	4.98E-17
IL5RA	-1.008	1.88E-18	5.11E-17
AF186192	-1.295	2.23E-18	6.02E-17
CPEB1	-1.116	2.42E-18	6.46E-17
CELA3A	-1.301	2.74E-18	7.28E-17
UTS2B	-1.107	3.27E-18	8.64E-17
CLEC4F	-1.062	3.27E-18	8.65E-17
IGKV7	-1.122	3.93E-18	1.03E-16
FOXI2	-1.258	4.13E-18	1.08E-16
RPL7AP28	-1.333	5.17E-18	1.35E-16
LILRP2	-1.142	6.02E-18	1.56E-16
IGHV1OR15	-1.269	6.76E-18	1.75E-16
IGSF11	-1.372	7.34E-18	1.89E-16

RGS22	-1.048	8.44E-18	2.15E-16
LRRTM4	-1.145	1.00E-17	2.55E-16
KCNC1	-1.085	1.12E-17	2.83E-16
AF064860	-1.349	1.20E-17	3.01E-16
CYP8B1	-1.240	1.39E-17	3.47E-16
TRGV4	-1.201	1.52E-17	3.79E-16
BRINP2	-1.197	1.66E-17	4.10E-16
SNAP25	-1.080	1.82E-17	4.50E-16
KRT14	-1.110	2.24E-17	5.50E-16
CETN4P	-1.333	2.40E-17	5.87E-16
NCAN	-1.303	2.67E-17	6.53E-16
TRGV2	-1.255	3.19E-17	7.76E-16
ANXA8L1	-1.011	4.03E-17	9.64E-16
DDX25	-1.263	4.42E-17	1.05E-15
AC002511	-1.004	5.13E-17	1.22E-15
SEC14L6	-1.008	5.85E-17	1.39E-15
FAM187B2P	-1.208	8.26E-17	1.94E-15
HCN4	-1.176	1.08E-16	2.52E-15
SFTPD	-1.062	1.09E-16	2.54E-15
hsa	-1.349	1.37E-16	3.18E-15
AC004637	-1.325	1.45E-16	3.35E-15
CCDC169	-1.324	1.57E-16	3.62E-15
MIR133A1HG	-1.227	2.09E-16	4.79E-15
TRIM50	-1.205	2.95E-16	6.71E-15
STAC2	-1.002	3.40E-16	7.68E-15
HCG4	-1.114	3.64E-16	8.18E-15
TPPP2	-1.214	4.38E-16	9.83E-15
PCDH8	-1.085	4.58E-16	1.03E-14
CARTPT	-1.033	5.43E-16	1.21E-14
LRP1B	-1.097	5.80E-16	1.29E-14
TRPC7	-1.143	6.39E-16	1.41E-14
AC246787	-1.251	7.00E-16	1.54E-14
PAX4	-1.095	7.40E-16	1.63E-14
CYP4F24P	-1.186	7.65E-16	1.68E-14
TMEM155	-1.014	8.77E-16	1.92E-14
WDR64	-1.288	1.42E-15	3.06E-14
GRIK1	-1.049	2.64E-15	5.60E-14
EFCAB1	-1.019	2.84E-15	6.00E-14
IGKV2	-1.150	3.13E-15	6.58E-14
GP9	-1.126	3.14E-15	6.60E-14
TCF23	-1.058	3.86E-15	8.05E-14
KIR3DL2	-1.290	4.33E-15	8.97E-14
AMHR2	-1.234	4.72E-15	9.73E-14
HIGD1AP1	-1.174	5.34E-15	1.09E-13

NPPC	-1.080	6.14E-15	1.24E-13
ZNF812	-1.142	6.52E-15	1.32E-13
ZFR2	-1.130	1.13E-14	2.25E-13
FAM216B	-1.041	1.30E-14	2.55E-13
SYT15	-1.038	1.36E-14	2.68E-13
MAP1LC3C	-1.032	1.39E-14	2.73E-13
GCSAML	-1.002	1.42E-14	2.77E-13
AC016995	-1.064	1.45E-14	2.83E-13
WFIKKN2	-1.029	1.62E-14	3.14E-13
SLC7A3	-1.167	1.65E-14	3.19E-13
IGLL1	-1.055	1.67E-14	3.24E-13
AL161668	-1.039	1.87E-14	3.61E-13
C1QTNF9	-1.229	2.21E-14	4.24E-13
CAMP	-1.097	2.55E-14	4.87E-13
OPN4	-1.184	3.04E-14	5.77E-13
TRGV3	-1.089	3.15E-14	5.96E-13
TRBV27	-1.226	3.19E-14	6.03E-13
IGLL3P	-1.202	3.23E-14	6.09E-13
C16orf96	-1.176	3.49E-14	6.57E-13
RIMS2	-1.034	3.99E-14	7.46E-13
TRIM63	-1.106	4.25E-14	7.93E-13
IGKV2D	-1.163	4.27E-14	7.95E-13
ELL2P1	-1.045	5.02E-14	9.28E-13
CCNA1	-1.074	5.38E-14	9.94E-13
PCDHA1	-1.173	1.14E-13	2.03E-12
SYT6	-1.068	1.21E-13	2.14E-12
CHODL	-1.002	1.37E-13	2.41E-12
UGT3A2	-1.059	1.55E-13	2.72E-12
LGSN	-1.057	1.87E-13	3.26E-12
UGT1A7	-1.128	2.52E-13	4.35E-12
ABHD17AP6	-1.186	6.02E-13	1.02E-11
GSTA2	-1.009	1.13E-12	1.86E-11
PEBP4	-1.116	1.14E-12	1.88E-11
GPR31	-1.108	2.05E-12	3.31E-11
DCX	-1.015	2.22E-12	3.57E-11
C5orf17	-1.009	3.79E-12	5.95E-11
LRRC37A7P	-1.051	4.49E-12	7.01E-11
DIRC3	-1.069	4.69E-12	7.30E-11
PKD1L2	-1.076	9.23E-12	1.39E-10
CNTN5	-1.074	9.44E-12	1.42E-10
AC078941	-1.006	1.28E-11	1.90E-10
RPL7P60	-1.084	1.89E-11	2.78E-10
BFSP2	-1.015	4.47E-11	6.30E-10
AC013264	-1.111	4.90E-11	6.87E-10

ANKRD20A4	-1.040	6.87E-11	9.48E-10
CALHM1	-1.015	7.04E-11	9.69E-10
SLC4A1	-1.073	1.74E-10	2.30E-09
AC005235	-1.063	2.22E-10	2.90E-09
PVALB	-1.010	2.71E-10	3.50E-09
HDAC1P1	-1.031	3.11E-10	3.99E-09

Table S3. Unfavorable genes in colon cancer

Gene names	UniProtKB	Length	Protein names
LRCH4	O75427	683	Leucine-rich repeat and calponin homology domain-containing protein 4
POFUT2	Q9Y2G5	429	GDP-fucose protein O-fucosyltransferase 2
CLK3	P49761	638	Dual specificity protein kinase CLK3
EGFL7	Q9UHF1	273	Epidermal growth factor-like protein 7
DPP7	Q9UHL4	492	Dipeptidyl peptidase 2
HSH2D	Q96JZ2	352	Hematopoietic SH2 domain-containing protein
ASB6	Q9NWX5	421	Ankyrin repeat and SOCS box protein 6
SPAG4	Q9NPE6	437	Sperm-associated antigen 4 protein
EXOC3L4	Q17RC7	722	Exocyst complex component 3-like protein 4
HSPA1A	P0DMV8	641	Heat shock 70 kDa protein 1A
PAQR6	Q6TCH4	344	Membrane progestin receptor delta
FAM69B	Q5VUD6	431	Divergent protein kinase domain 1B
CRACR2B	Q8N4Y2	399	EF-hand calcium-binding domain-containing protein 4A
ARHGAP4	P98171	946	Rho GTPase-activating protein 4
NPDC1	Q9NQX5	325	Neural proliferation differentiation and control protein 1
DAPK1	P53355	1430	Death-associated protein kinase 1
CNPY3	Q9BT09	278	Protein canopy homolog 3
ARL8A	Q96BM9	186	ADP-ribosylation factor-like protein 8A
INAFM1	C9JVW0	142	Putative transmembrane protein INAFM1
RHBDD2	Q6NTF9	364	Rhomboid domain-containing protein 2
SEMA4C	Q9C0C4	833	Semaphorin-4C
GDI1	P31150	447	Rab GDP dissociation inhibitor alpha
SLC10A3	P09131	477	P3 protein
PI4K2A	Q9BTU6	479	Phosphatidylinositol 4-kinase type 2-alpha
HOMER3	Q9NSC5	361	Homer protein homolog 3
HSD17B14	Q9BPX1	270	17-beta-hydroxysteroid dehydrogenase 14
NPC2	P61916	151	NPC intracellular cholesterol transporter 2
CRYAB	P02511	175	Alpha-crystallin B chain
LIMK1	P53667	647	LIM domain kinase 1
PYCR2	Q96C36	320	Pyrroline-5-carboxylate reductase 2
RRNAD1	Q96FB5	475	Protein RRNAD1
CALB2	P22676	271	Calretinin
GPC1	P35052	558	Glypican-1 [Cleaved into: Secreted glypican-1]
VAMP1	P23763	118	Vesicle-associated membrane protein 1

PPP1R3E	Q9H7J1	279	Protein phosphatase 1 regulatory subunit 3E
HOXD13	P35453	343	Homeobox protein Hox-D13
ALAD	P13716	330	Delta-aminolevulinic acid dehydratase
ADARB1	P78563	741	Double-stranded RNA-specific editase 1
HSF4	Q9ULV5	492	Heat shock factor protein 4
ARHGEF17	Q96PE2	2063	Rho guanine nucleotide exchange factor 17
JDP2	Q8WYK2	163	Jun dimerization protein 2
MGEA5	O60502	916	Protein O-GlcNAcase
COMP	P49747	757	Cartilage oligomeric matrix protein
AMH	P03971	560	Muellerian-inhibiting factor
GPRC5B	Q9NZH0	403	G-protein coupled receptor family C group 5 member B
KCNE4	Q8WWG9	221	Potassium voltage-gated channel subfamily E member 4
FAM50A	Q14320	339	Protein FAM50A
TMEM106A	Q96A25	262	Transmembrane protein 106A
PHF1	O43189	567	PHD finger protein 1
PRELP	P51888	382	Prolargin
MTHFSD	Q2M296	383	Methenyltetrahydrofolate synthase domain-containing protein
HSPB1	P04792	205	Heat shock protein beta-1
AGAP9	Q5VTM2	703	Arf-GAP with GTPase, ANK repeat and PH domain-containing protein 9
DNAJB2	P25686	324	DnaJ homolog subfamily B member 2
LMBR1L	Q6UX01	489	Protein LMBR1L
WBP1	Q96G27	269	WW domain-binding protein 1
CYHR1	Q6ZMK1	362	Cysteine and histidine-rich protein 1
IGFBP3	P17936	291	Insulin-like growth factor-binding protein 3
FAM167B	Q9BTA0	163	Protein FAM167B
TMEM184A	Q6ZMB5	413	Transmembrane protein 184A
TMEM88	Q6PEY1	159	Transmembrane protein 88
ALDH3B1	P43353	468	Aldehyde dehydrogenase family 3 member B1
FKBP8	Q14318	412	Peptidyl-prolyl cis-trans isomerase FKBP8
S1PR3	Q99500	378	Sphingosine 1-phosphate receptor 3
GATAD2B	Q8WXI9	593	Transcriptional repressor p66-beta
SEZ6L2	Q6UXD5	910	Seizure 6-like protein 2
WWC3	Q9ULE0	1092	Protein WWC3
THAP3	Q8WTV1	239	THAP domain-containing protein 3
VSIG4	Q9Y279	399	V-set and immunoglobulin domain-containing protein 4
TBC1D10B	Q4KMP7	808	TBC1 domain family member 10B
DENND6B	Q8NEG7	585	Protein DENND6B
INHBB	P09529	407	Inhibin beta B chain
GAS1	P54826	345	Growth arrest-specific protein 1
GPR137B	O60478	399	Integral membrane protein GPR137B
GOLGA2	Q08379	1002	Golgin subfamily A member 2
CRY2	Q49AN0	593	Cryptochrome-2

DEF6	Q01524	100	Defensin-6
WBSCR27	Q8N6F8	245	Methyltransferase-like protein 27
FAM219B	Q5XKK7	198	Protein FAM219B
FAM3A	P98173	230	Protein FAM3A
CCM2	Q9BSQ5	444	Cerebral cavernous malformations 2 protein
RHOG	P84095	191	Rho-related GTP-binding protein RhoG
ADAMTSL4	Q6UY14	1074	ADAMTS-like protein 4
DVL3	Q92997	716	Segment polarity protein dishevelled homolog DVL-3
SPOCK1	Q08629	439	Testican-1
AIP	O00170	330	AH receptor-interacting protein
NR1D1	P20393	614	Nuclear receptor subfamily 1 group D member 1
TIMP1	P01033	207	Metalloproteinase inhibitor 1
RNF32	Q9H0A6	362	RING finger protein 32
PNMA5	Q96PV4	448	Paraneoplastic antigen-like protein 5
ANKRD39	Q53RE8	183	Ankyrin repeat domain-containing protein 39
BCAM	P50895	628	Basal cell adhesion molecule
PTGES	O14684	152	Prostaglandin E synthase
KRTAP5-1	Q6L8H4	278	Keratin-associated protein 5-1
TNS1	Q9HBL0	1735	Tensin-1
THBS3	P49746	956	Thrombospondin-3
CHD3	Q12873	2000	Chromodomain-helicase-DNA-binding protein 3
CTSD	P07339	412	Cathepsin D
USP20	Q9Y2K6	914	Ubiquitin carboxyl-terminal hydrolase 20
CUL9	Q8IWT3	2517	Cullin-9
RBP7	Q96R05	134	Retinoid-binding protein 7
TAOK2	Q9UL54	1235	Serine/threonine-protein kinase TAO2
SLC39A13	Q96H72	371	Zinc transporter ZIP13
TPM2	P07951	284	Tropomyosin beta chain
TSC22D4	Q9Y3Q8	395	TSC22 domain family protein 4
PBXIP1	Q96AQ6	731	Pre-B-cell leukemia transcription factor-interacting protein 1
KDM4B	O94953	1096	Lysine-specific demethylase 4B
SERPINE1	P05121	402	Plasminogen activator inhibitor 1
RNF187	Q5TA31	235	E3 ubiquitin-protein ligase RNF187
AFAP1L1	Q8TED9	768	Actin filament-associated protein 1-like 1
MIER2	Q8N344	545	Mesoderm induction early response protein 2
HIST2H2BE	Q16778	126	Histone H2B type 2-E
PRNP	F7VJQ1	73	Alternative prion protein
C8G	P07360	202	Complement component C8 gamma chain
FSTL3	O95633	263	Follistatin-related protein 3
OLFM2	O95897	454	Noelin-2
MEGF6	O75095	1541	Multiple epidermal growth factor-like domains protein 6
DNAJC4	Q9NNZ3	241	DnaJ homolog subfamily C member 4
IDUA	P35475	653	Alpha-L-iduronidase

PPP2R2D	Q66LE6	453	Serine/threonine-protein phosphatase 2A 55 kDa regulatory subunit B delta isoform
TSPYL2	Q9H2G4	693	Testis-specific Y-encoded-like protein 2
PLOD3	O60568	738	Multifunctional procollagen lysine hydroxylase and glycosyltransferase LH3
PARP6	Q2NL67	630	Protein mono-ADP-ribosyltransferase PARP6
NUMBL	Q9Y6R0	609	Numb-like protein
AKAP8L	Q9ULX6	646	A-kinase anchor protein 8-like
PLEKHG2	Q9H7P9	1386	Pleckstrin homology domain-containing family G member 2
FOXC1	Q12948	553	Forkhead box protein C1
TRIP10	Q15642	601	Cdc42-interacting protein 4
PXDC1	Q5TGL8	231	PX domain-containing protein 1
TMPRSS3	P57727	454	Transmembrane protease serine 3
PRKRIP1	Q9H875	184	PRKR-interacting protein 1
AOC3	Q16853	763	Membrane primary amine oxidase
SCT	P09683	121	Secretin
CBX7	O95931	251	Chromobox protein homolog 7
LMNTD2	Q8IXW0	634	Lamin tail domain-containing protein 2
CLK2	P49760	499	Dual specificity protein kinase CLK2
PABPC1L	Q4VXU2	614	Polyadenylate-binding protein 1-like
HIST1H2BD	P58876	126	Histone H2B type 1-D
NFKB2	Q00653	900	Nuclear factor NF-kappa-B p100 subunit
PPP1R26	Q5T8A7	1209	Protein phosphatase 1 regulatory subunit 26
SPARCL1	Q14515	664	SPARC-like protein 1
PLEC	Q15149	4684	Plectin
EPHB4	P54760	987	Ephrin type-B receptor 4
ENDOV	Q8N8Q3	282	Endonuclease V
EPS8L1	Q8TE68	723	Epidermal growth factor receptor kinase substrate 8-like protein 1
TNFRSF25	Q93038	417	Tumor necrosis factor receptor superfamily member 25
PCGF2	P35227	344	Polycomb group RING finger protein 2
SHC2	P98077	582	SHC-transforming protein 2
TNNT1	P13805	278	Troponin T, slow skeletal muscle
PPP1R18	Q6NYC8	613	Phostensin
FASTK	Q14296	549	Fas-activated serine/threonine kinase
SNAI1	O95863	264	Zinc finger protein SNAI1
DNAJB12	Q9NXW2	375	DnaJ homolog subfamily B member 12
HYI	Q5T013	277	Putative hydroxypyruvate isomerase
ADAMTSL2	Q86TH1	951	ADAMTS-like protein 2
MFNG	O00587	321	Beta-1,3-N-acetylglucosaminyltransferase manic fringe
TFPT	P0C1Z6	253	TCF3 fusion partner
ZNF385A	Q96PM9	386	Zinc finger protein 385A
ADAP1	O75689	374	Arf-GAP with dual PH domain-containing protein 1
ABHD8	Q96I13	439	Protein ABHD8

MOGS	Q13724	837	Mannosyl-oligosaccharide glucosidase
PTPN6	P29350	595	Tyrosine-protein phosphatase non-receptor type 6
GAL3ST4	Q96RP7	486	Galactose-3-O-sulfotransferase 4
TMEM185B	Q9H7F4	350	Transmembrane protein 185B
COQ10A	Q96MF6	247	Coenzyme Q-binding protein COQ10 homolog A, mitochondrial
SMARCD3	Q6STE5	483	SWI/SNF-related matrix-associated actin-dependent regulator of chromatin subfamily D member 3
UBTD1	Q9HAC8	227	Ubiquitin domain-containing protein 1
TLN1	Q9Y490	2541	Talin-1
NEXN	Q0ZGT2	675	Nexilin
RGCC	Q9H4X1	137	Regulator of cell cycle RGCC
ADAP2	Q9NPF8	381	Arf-GAP with dual PH domain-containing protein 2
CRTC2	Q53ET0	693	CREB-regulated transcription coactivator 2
FES	P07332	822	Tyrosine-protein kinase Fes/Fps
FLOT1	O75955	427	Flotillin-1
NFATC4	Q14934	902	Nuclear factor of activated T-cells, cytoplasmic 4
DNAJC17	Q9NVM6	304	DnaJ homolog subfamily C member 17
HSD3B7	Q9H2F3	369	3 beta-hydroxysteroid dehydrogenase type 7
STK25	O00506	426	Serine/threonine-protein kinase 25
GPSM1	Q86YR5	675	G-protein-signaling modulator 1
RAB3IL1	Q8TBN0	382	Guanine nucleotide exchange factor for Rab-3A
PHF21A	Q96BD5	680	PHD finger protein 21A
GBGT1	Q8N5D6	347	Globoside alpha-1,3-N-acetylgalactosaminyltransferase 1
PTRF	Q6NZI2	390	Caveolae-associated protein 1
PLPPR2	Q96GM1	343	Phospholipid phosphatase-related protein type 2
TOR4A	Q9NXH8	423	Torsin-4A
SOX13	Q9UN79	622	Transcription factor SOX-13
EBF4	Q9BQW3	602	Transcription factor COE4
PTOV1	Q86YD1	416	Prostate tumor-overexpressed gene 1 protein
RUBCN	Q92622	972	Run domain Beclin-1-interacting and cysteine-rich domain-containing protein
TPBG	Q13641	420	Trophoblast glycoprotein
TBC1D8	O95759	1140	TBC1 domain family member 8
BCL7A	Q4VC05	210	B-cell CLL/lymphoma 7 protein family member A
ATG4B	Q9Y4P1	393	Cysteine protease ATG4B
ZFYVE27	Q5T4F4	411	Protrudin
ENO2	P09104	434	Gamma-enolase
NEK8	Q86SG6	692	Serine/threonine-protein kinase Nek8
STAC3	Q96MF2	364	SH3 and cysteine-rich domain-containing protein 3
ABCA7	Q8IZY2	2146	Phospholipid-transporting ATPase ABCA7
TLX1	P31314	330	T-cell leukemia homeobox protein 1
ENO3	P13929	434	Beta-enolase
FLNA	P21333	2647	Filamin-A

SHANK3	Q9BYB0	1731	SH3 and multiple ankyrin repeat domains protein 3
PKD1	P98161	4303	Polycystin-1
ANGPTL4	Q9BY76	406	Angiopoietin-related protein 4
CD81	P60033	236	CD81 antigen
STAB1	Q9NY15	2570	Stabilin-1
RRAGB	Q5VZM2	374	Ras-related GTP-binding protein B
GAA	P10253	952	Lysosomal alpha-glucosidase
C1orf35	Q9BU76	263	Multiple myeloma tumor-associated protein 2
G0S2	P27469	103	G0/G1 switch protein 2
FAM134A	Q8NC44	543	Reticulophagy regulator 2
NRIP2	Q9BQI9	281	Nuclear receptor-interacting protein 2
KCTD7	Q96MP8	289	BTB/POZ domain-containing protein KCTD7
UCN	P55089	124	Urocortin
LZTS2	Q9BRK4	669	Leucine zipper putative tumor suppressor 2
HOXD9	P28356	352	Homeobox protein Hox-D9
BATF	Q16520	125	Basic leucine zipper transcriptional factor ATF-like
PLIN4	Q96Q06	1357	Perilipin-4
ZNF653	Q96CK0	615	Zinc finger protein 653
FOLR2	P14207	255	Folate receptor beta
ITGA5	P08648	1049	Integrin alpha-5
LCAT	P04180	440	Phosphatidylcholine-sterol acyltransferase
NR2C2AP	Q86WQ0	139	Nuclear receptor 2C2-associated protein
KCTD13	Q8WZ19	329	BTB/POZ domain-containing adapter for CUL3-mediated RhoA degradation protein 1
SLC2A3	P11169	496	Solute carrier family 2, facilitated glucose transporter member 3
SF3A2	Q15428	464	Splicing factor 3A subunit 2
RUNX3	Q13761	415	Runt-related transcription factor 3
OAZ2	O95190	189	Ornithine decarboxylase antizyme 2
KCNJ8	Q15842	424	ATP-sensitive inward rectifier potassium channel 8
TRAF2	Q12933	501	TNF receptor-associated factor 2
PHF2	O75151	1096	Lysine-specific demethylase PHF2
KRI1	Q8N9T8	703	Protein KRI1 homolog
SIPA1	Q96FS4	1042	Signal-induced proliferation-associated protein 1
CSAD	Q9Y600	493	Cysteine sulfinic acid decarboxylase
TUT1	Q9H6E5	874	Speckle targeted PIP5K1A-regulated poly
AGAP3	Q96P47	875	Arf-GAP with GTPase, ANK repeat and PH domain-containing protein 3
SCAF1	Q9H7N4	1312	Splicing factor, arginine/serine-rich 19
CLASRP	Q8N2M8	674	CLK4-associating serine/arginine rich protein
NR1H2	P55055	460	Oxysterols receptor LXR-beta
C9orf16	Q9BUW7	83	UPF0184 protein C9orf16
PHKG2	P15735	406	Phosphorylase b kinase gamma catalytic chain, liver/testis isoform

SERPING1	P05155	500	Plasma protease C1 inhibitor

Table S4. In all modules of WGCNA, the genes ranked in the top 1000 in connectivity

fromNode	toNode	weight
ABCC9	DDR2	0.28393497
AC018738	RPL41P1	0.318344881
AC026271	RPL41P1	0.287650075
AC107983	HNRNPCP2	0.347219912
ACSS3	DDR2	0.262884034
ACTC1	MSRB3	0.272329137
ACTG2	LMOD1	0.306304734
ADAM12	COL5A2	0.277567183
ADAMTS12	COL5A2	0.298899887
ADAMTS16	DDR2	0.277805673
ADAMTS2	COL3A1	0.283151826
ADCY2	MSRB3	0.276771399
AKAP12	DDR2	0.270114221
AL158801	RPL41P1	0.272151583
AMOTL1	DDR2	0.274032367
ANTXR1	DDR2	0.28602415
AOC3	CNN1	0.282239887
ARHGEF25	MSRB3	0.264466426
ASB5	LMOD1	0.263268793
ASPN	DDR2	0.298157265
BEND6	DDR2	0.274523683
BGN	COL1A2	0.291674197
BNC2	DDR2	0.282499264
BOC	MSRB3	0.262587294
BTF3L4P2	RPL41P1	0.278675854
C11orf98	RPL41P1	0.313183097
C20orf194	SGCD	0.268879679
CACNA2D1	DDR2	0.281039235
CALD1	DDR2	0.289600596
CARTPT	LMOD1	0.274913361
CASQ2	LMOD1	0.277796732
CCDC8	DDR2	0.269132005
CCDC80	DDR2	0.268863758
CD3G	IKZF1	0.272566082
CD79A	IGHJ3	0.288620599
CDH2	DDR2	0.289700785
CDK14	DDR2	0.271640276
CHCHD2P9	RPL41P1	0.318939837
CHRDL2	LMOD1	0.277457789
CHRM2	LMOD1	0.281077216

CLMP	DDR2	0.273467177
CNN1	LMOD1	0.295713673
COL10A1	SULF1	0.296699008
COL11A1	SULF1	0.296839853
COL12A1	FBN1	0.269049988
COL1A1	COL1A2	0.311159138
COL1A2	PPAPDC1A	0.287323169
COL24A1	DDR2	0.262213365
COL3A1	PPAPDC1A	0.284489342
COL5A2	PPAPDC1A	0.285482178
COL6A3	DDR2	0.267786672
COL8A1	CORIN	0.277262786
COL8A2	DDR2	0.270499579
COMP	THBS2	0.272014828
CORIN	SULF1	0.277585322
CPXM2	MSRB3	0.265523688
CRISPLD1	DDR2	0.282331425
CRYAB	LMOD1	0.261519028
CSDC2	MSRB3	0.277465041
CTAGE8	RPL41P1	0.27966984
CTHRC1	SULF1	0.288265066
CXCR2P1	IKZF1	0.266589011
CYP27C1	DDR2	0.269529323
CYP7B1	DDR2	0.264748738
DCN	DDR2	0.261814596
DDR2	SGCD	0.297013854
DDT	RPL41P1	0.275469477
DES	LMOD1	0.306551002
DGKI	MSRB3	0.265299952
DKK2	FBN1	0.266865489
DPYSL3	MSRB3	0.274032222
EEF1A1P16	RPL6P27	0.28238306
EEF1A1P6	HNRNPCP2	0.331056042
EEF1B2P6	RPL6P27	0.342943793
EEF1G	RPL41P1	0.308771101
EIF4BP3	RPL41P1	0.263048004
EIF4BP6	HNRNPCP2	0.297702396
EIF4BP7	RPL41P1	0.302502284
EVC	MSRB3	0.264778878
FABP5P7	HNRNPCP2	0.287412204
FAM180A	MSRB3	0.274821484
FAM92B	IGHV3	0.278847327
FAP	SULF1	0.275440723
FBN1	ITGA11	0.280509336

FGF1	MSRB3	0.274427527
FIBIN	MSRB3	0.28272229
FLNA	TNS1	0.262443312
FLRT2	MSRB3	0.264762482
FST	MSRB3	0.271093449
GBP5	PTPRC	0.266078819
GDF5	LMOD1	0.276533795
GDF6	MSRB3	0.26669247
GLI3	MSRB3	0.273755954
GLRB	MSRB3	0.282552368
GNAO1	HAND1	0.273579154
GREM1	MSRB3	0.267053962
GTF2IP1	HNRNPA1P38	0.266084151
GUCY1A3	MSRB3	0.281209235
HAND1	LMOD1	0.307465138
HAND2	MSRB3	0.268903131
HIST1H2AB	HIST1H4C	0.263212959
HIST1H2AH	HIST1H4C	0.264904105
HIST1H3I	HIST1H4C	0.263317065
HIST1H4A	HIST1H4C	0.26269903
HIST1H4B	HIST1H4C	0.268327915
HMGB1P10	RPL41P1	0.307147171
HMGB1P5	HNRNPA1P38	0.309882851
HMGN1P37	RPL41P1	0.291073368
HMGN2P17	RPL41P1	0.29255908
HMGN2P3	RPL41P1	0.324351272
HMGN2P41	RPL41P1	0.329462886
HNRNPA1P10	HNRNPCP2	0.309048406
HNRNPA1P38	RPL41P1	0.342180459
HNRNPCP2	RPL35P1	0.341861466
HOXB5	HOXB6	0.336688595
HOXB6	HOXB8	0.329970781
HOXB8	HOXB9	0.266303066
HSPB6	LMOD1	0.268577364
HSPB7	LMOD1	0.293227231
HSPB8	MSRB3	0.263443577
IFNWP19	MIR31HG	0.298076444
IGFBP5	MSRB3	0.273042202
IGHA1	IGHV3	0.333031668
IGHA2	IGHV3	0.281741868
IGHG2	IGKV1	0.277392957
IGHG4	IGKV1	0.291867493
IGHJ1	IGHV3	0.365425495
IGHJ2	IGHV3	0.399820121

IGHJ3	IGHV3	0.405914847
IGHM	IGHV3	0.277765637
IGHV1	IGLC2	0.343472762
IGHV1OR21	IGHV3	0.291250938
IGHV2	IGLC2	0.278493482
IGHV3	IGLC2	0.364506351
IGHV3OR15	IGKV1	0.279832655
IGHV4	IGLC2	0.341359654
IGHV5	IGLC2	0.286208235
IGHV6	IGKV1	0.277716281
IGJ	IGKV1	0.265331767
IGKC	IGLC2	0.315162526
IGKJ5	IGKV1	0.337604936
IGKV1	IGLC2	0.362930611
IGKV2OR22	IGLV1	0.28889708
IGKV4	IGLV1	0.304432248
IGKV6	IGLV1	0.267582133
IGKV7	IGLV1	0.299606167
IGLC2	IGLV1	0.3388089
IGLC3	IGLV1	0.290989675
IGLC7	IGLV1	0.287793824
IGLL1	IGLV1	0.311130561
IGLL5	IGLV1	0.301152999
IGLV1	IGLV6	0.317745316
IGLV2	IGLV6	0.299013986
IGLV4	MZB1	0.269218665
IGLV6	MZB1	0.294121639
IGLV8	MZB1	0.273469489
IGLV9	MZB1	0.276461469
IKZF1	SH2D1A	0.273646452
JPH2	LMOD1	0.272437755
KAL1	SULF1	0.274238703
KCNMB1	LMOD1	0.272610108
KIAA1462	MSRB3	0.262130208
KIF26B	SULF1	0.266041728
KLHL4	MSRB3	0.262768929
LIMS2	LMOD1	0.264774686
LMOD1	MORN5	0.286902283
LRRC4C	MSRB3	0.268897032
LSM12P1	RPL41P1	0.305330649
